# IFNg_DeepKG: A Novel Model for Identifying Interferon-Gamma-Inducing Epitopes Using Knowledge Graph RAG in Biomedical Applications

**DOI:** 10.1021/acs.jcim.5c02248

**Published:** 2025-12-31

**Authors:** Van The Le, Juan Peter Timothy Yuune, Yu-Yen Ou

**Affiliations:** † Department of Computer Science and Engineering, 34895 Yuan Ze University, Chung-Li, Taoyuan City 32003, Taiwan; ‡ Graduate Program in Biomedical Informatics, Yuan Ze University, Chung-Li, Taoyuan City 32003, Taiwan

## Abstract

The accurate and efficient computational identification of interferon-gamma-inducing epitopes (IFNgIE) is a critical bottleneck in the design of next-generation vaccines and immunotherapies. Existing computational models, while adept at learning sequence-based patterns, frequently fail to incorporate the rich biological context that governs an epitope’s immunogenicity, such as its protein of origin, host, and disease association. To address this limitation, we propose IFNg_DeepKG, a new deep learning framework that synergistically integrates a pretrained protein language model (ESM2), a custom knowledge graph (KG) using a Retrieval-Augmented Generation (RAG) approach, and a multiscale convolutional neural network (MSCNN). The model’s central innovation lies in its use of the RAG-KG to enrich sequence embeddings with external, biologically informed context, thereby significantly enhancing predictive performance. IFNg_DeepKG demonstrates superior performance on independent test data sets, achieving an AUC of 0.99 on the Human H_IFNgInd1 data set and 0.95 on the Mouse M_IFNgInd1 data set, a substantial increase over baseline models. With the more challenging independent data sets, the model demonstrated strong cross-species generalization, achieving AUCs of 0.94 (H_IFNgInd2) and 0.93 (M_IFNgInd2). The framework successfully identifies and classifies clinically relevant epitopes, including those associated with COVID-19 and Alzheimer’s disease. By bridging the gap between sequence-based features and biological contexts, IFNg_DeepKG represents a significant advancement in computational immunology, offering a scalable and powerful platform for rational epitope discovery and precision medicine.

## Graphical abstract

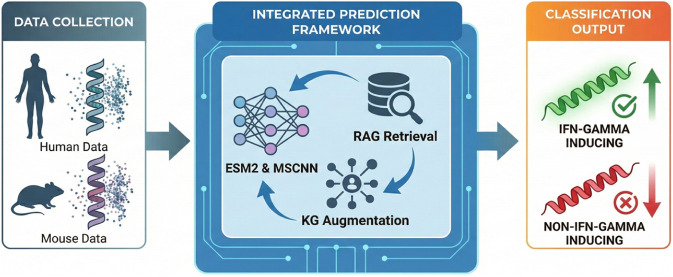

## 1 Introduction

Interferon-gamma (IFN-gamma) is a pleiotropic cytokine of paramount importance in the immune system, orchestrating both innate and adaptive immune responses. As depicted in Figure 1, its functions include the activation of macrophages, enhancement of antimicrobial activity, and promotion of T-cell activation, which collectively enable a robust immune response against pathogens, tumors, and other threats. IFN-gamma-inducing epitopes (IFNgIE) are short peptide sequences, typically 8 to 20 amino acids long, presented by major histocompatibility complex (MHC) molecules to activate T-cells, particularly CD8+ cytotoxic T-lymphocytes and CD4+ helper T-cells. The identification of these epitopes is essential for a wide range of biological and clinical applications.

**Figure 1:**
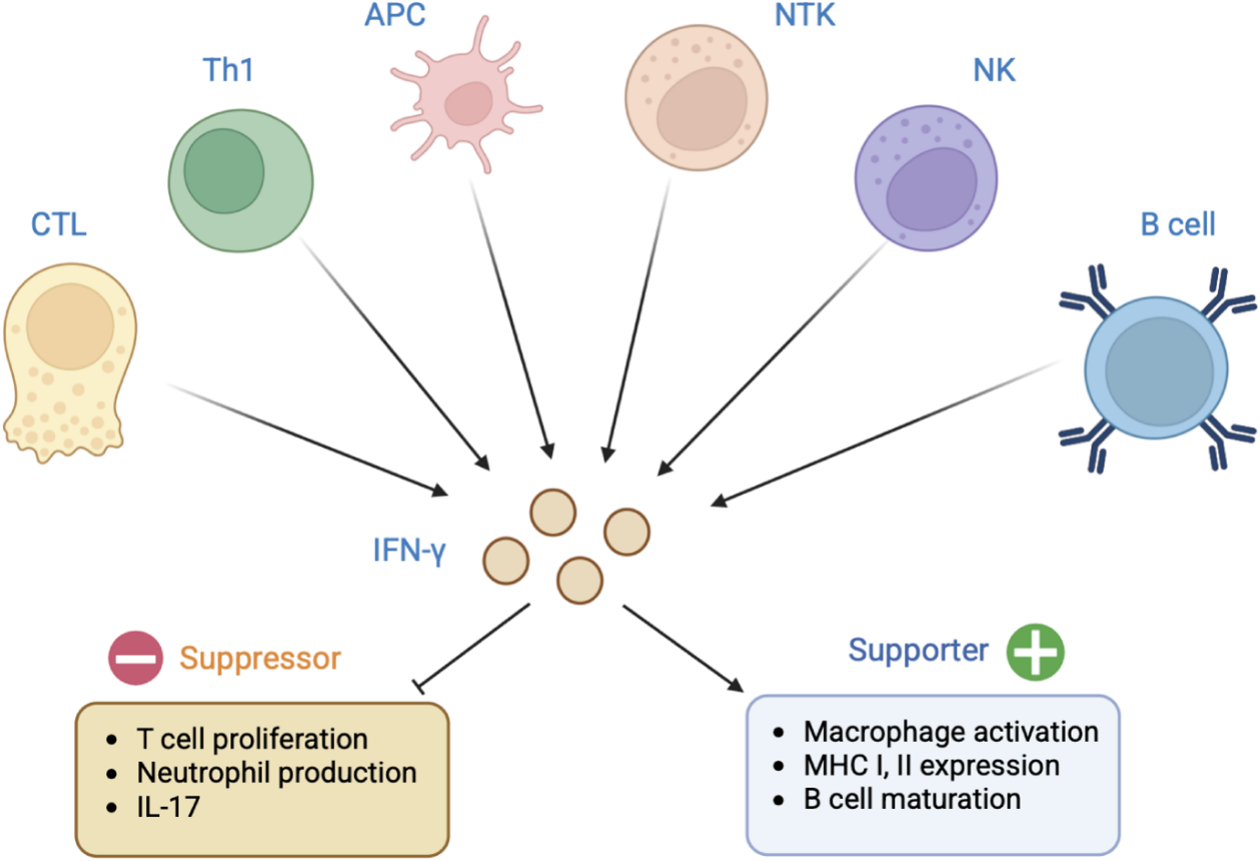
IFN-gamma plays a central role in orchestrating immune responses by enhancing antigen presentation, activating macrophages, and promoting Th1 effector functions. It supports B cell maturation and antibody production, while simultaneously suppressing Th17 activity, IL-17 secretion, and excessive neutrophil responses. These diverse functions highlight its importance in protective immunity and its potential applications in vaccination strategies against infectious diseases (e.g., COVID-19) and immune-mediated conditions (e.g., diabetes, Alzheimer’s disease).

In modern medicine, IFNgIEs are central to the development of peptide-based vaccines for infectious diseases, such as tuberculosis and HIV, as well as for cancer.^1–4^ The ability to precisely identify immunogenic epitopes allows for the rational design of vaccines that target specific, highly effective components of a pathogen or tumor, thereby eliciting a more potent and focused immune response. Beyond vaccines, IFNgIEs are pivotal in engineering T-cell therapies, such as chimeric antigen receptor (CAR) T-cells, to enhance antitumor immunity by directing T-cells to specific cancer antigens. Recent research has further solidified the link between IFN-gamma and the success of modern immunotherapies, noting that immune checkpoint blockade therapies, which have achieved notable benefits in a wide variety of cancers, act at least in part by increasing IFN-gammaproduction. Conversely, resistance to these therapies has been attributed to defects in the IFN-gammasignaling pathway, underscoring the cytokine’s critical role in cancer immunity.^5^ Given their vast therapeutic potential, IFNgIEs are promising candidates for advancing precision medicine.

Recent biological studies have advanced our understanding of IFNgIEs. First, high-throughput assays, such as ELISpot^6^ and MHC tetramer staining,^7^ identify epitopes by measuring IFN-gamma secretion in T-cell assays. For example, studies on *Mycobacterium tuberculosis* have identified epitopes like ESAT-6 as potent IFN-gamma inducers.^8,9^ Moreover, advances in peptide synthesis technologies allow the production of custom epitopes for therapeutic testing, enabling rapid prototyping of peptide-based vaccines.^10–14^ These methods provide beneficially high specificity and direct biological insights into epitope function. Despite their advantages, experimental approaches are labor-intensive, costly, and limited by the need for patient-specific MHC typing and low throughput.

To overcome the drawbacks of experiment-based studies, computational methods have aided in accelerating epitope identification. Early approaches, such as those relying on Support Vector Machines (SVMs), often used hand-crafted features like amino acid composition (AAC) and dipeptide composition (DPC).^15,16^ In the computational field, tools like NetMHCpan^17^ and DeepImmuno^18^ use sequence-based features to predict MHC binding and immunogenicity. Moreover, TransPHLA,^19^ CapHLA,^20^ and BigMHC^21^ represent the current state-of-the-art deep learning systems for prediction of peptide-MHC binding, presentation, and immunogenicity across both MHC class I and II. However, they often focus on binding site prediction rather than the direct classification of IFN-gamma-inducing peptides. Although effective in certain contexts, these methods face limitations such as feature redundancy and limited biological relevance. Further exploration is necessary to enhance prediction accuracy and uncover key sequence features underlying IFN-gamma inducers.

More recent models have leveraged advances in artificial intelligence, incorporating embeddings from pretrained protein language models (PLMs) like ProtTrans^22^ and ESM2 ^23^ to represent amino acid sequences. These models have demonstrated improved performance by capturing more nuanced contextual information from the sequence itself. However, a significant limitation persists: most models rely solely on sequence data and fail to incorporate the broader biological context in which epitopes function, such as their source protein, host organism, or associated diseases. This lack of contextual awareness can limit the models’ generalizability and lead to suboptimal performance, particularly for complex immune interactions.

Exterior knowledge metadata can address this by encoding relationships between epitopes, proteins, organisms, and diseases. Recently, there are many ways to build a strict knowledgeable database to support the training process with protein/peptide samples. By integrating a knowledge graph with a Pretrained Language Model-based deep learning model (such as ESM2), we can capture both sequence motifs and biological context, improving prediction accuracy and enabling therapeutic applications in peptide-based drug design.

This study presents a novel framework, IFNg_DeepKG, designed to bridge the gap between computational efficiency and biological relevance. The approach combines a state-of-the-art pretrained language model with a biologically informed knowledge graph (KG) in a Retrieval-Augmented Generation (RAG) framework. This KG encodes complex relationships between epitopes, proteins, organisms, and diseases, allowing the model to incorporate functional and biological relevance in addition to sequence similarity. By doing so, IFNg_DeepKG moves beyond a purely data-driven approach, providing a model that is both highly accurate and more biologically interpretable.

## 2 Materials and Methods

Figure 2 describes our workflow for this study. It comprises 4 main sections. Section 1: Data collection from the IEDB database for updated IFN-gamma-inducing and noninducing peptides. These data are used for the Knowledge Graph RAG database. Also, training and independent testing data are reused from Dhall et al.^15^ Section 2: Pretrained Language Model using the ESM2 model. In this step, we convert all FASTA peptide sequences to two-dimensional profiles Lx1280, where L is the length of each peptide, while 1280 is the ESM2 dimension size. Section 3: Knowledge Graph RAG fusion. In this step, we combine each query sequence in the training and testing data with the Knowledge Graph RAG database to produce a new, better embedding for that query. This query was fused with similar peptide sequences sharing the same relationships in the Knowledge Graph map. Section 4: Prediction with MSCNN architecture. In this final step, we use MSCNN with various windows to parallely scan the whole sequences and learn their motifs.

**Figure 2:**
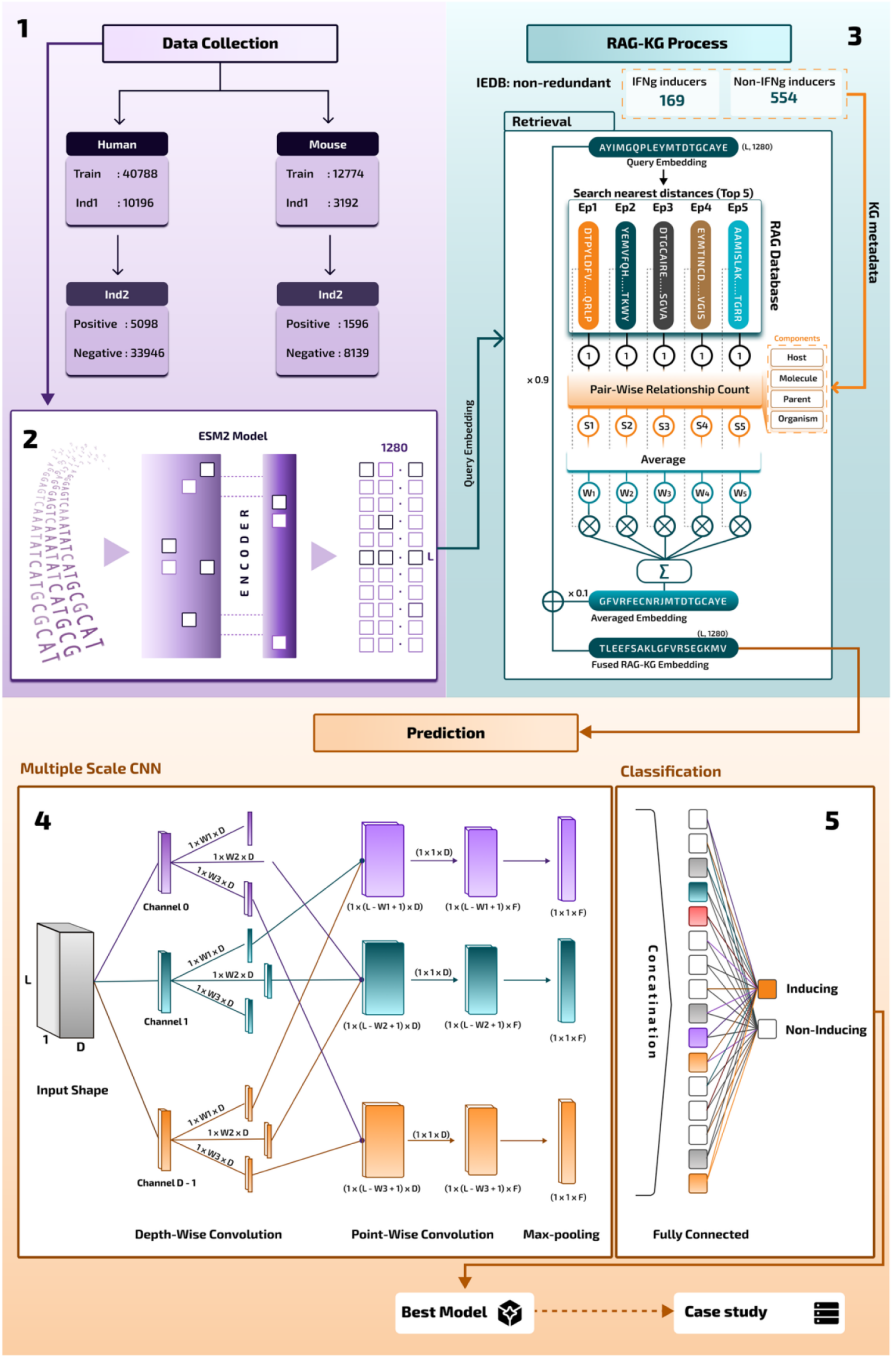
Overall workflow of the proposed framework. Panel 1: Data collection and preprocessing. Peptide sequences were gathered from publicly available databases and curated to ensure nonredundancy and balanced class distribution. Panel 2: Embedding generation.Each peptide sequence was transformed into high-dimensional feature representations using the ESM2 protein language model, capturing semantic and structural information from the amino acid context. Panel 3: Knowledge Graph–augmented Retrieval-Augmented Generation (RAG–KG) integration. The query embedding retrieves its five most relevant knowledge-enhanced embeddings from the RAG database, leveraging prior biological knowledge and relational information among peptides. Panel 4: Feature extraction and model training. A Multi-Scale Convolutional Neural Network (MSCNN) processes the integrated embeddings to capture both local motif-level and global contextual features. Panel 5: Classification stage. The final output layer predicts whether each peptide sequence is IFN-gamma-inducing or noninducing, completing the end-to-end workflow of the proposed framework.

Our proposed MSCNN model demonstrated strong and consistent predictive performance across multiple independent evaluations. On Human Independent Data set 1, the model achieved an AUC of 0.9878, while on Mouse Independent Data set 1, it reached an AUC of 0.9510. When further assessed on the more challenging and generalized Independent Data set 2, the model maintained solid performance with an AUC of 0.9449 for human data and an AUC of 0.9309 for mouse data, highlighting its strong generalization ability across species and data sets.

### 2.1 Data Collection

In Dhall et al.’s study,^15^ experimentally validated IFN-gamma-inducing and noninducing peptides were retrieved from the Immune Epitope Database (IEDB).^24^ IEDB provides high-quality, curated data with experimental validation, ensuring reliability. Peptides containing non-natural amino acids were excluded.

Host-specific filtering showed that most sequences originated from human and mouse hosts. Therefore, only these were retained for further analysis. The authors then filtered peptides by length, selecting those between 8 and 20 amino acids, as this range encompasses the majority of experimentally validated IFN-gamma epitopes in IEDB and corresponds to the typical lengths of MHC class I (8–11 residues) and class II (13–20 residues) peptides. Sequences shorter than 8 or longer than 20 residues were discarded to avoid incomplete or nonstandard entries. Redundant sequences were removed within each host-specific data set. In particular, noninducing peptides that either contained IFN-gamma-inducing peptides or differed by only one or two amino acids were excluded to minimize sequence redundancy.^15^

After preprocessing, the data sets were divided into Training Data set and Independent Data set 1 following an 80:20 ratio for each host. In our study, Training and Independent Data set 1 are denoted as H_IFNgTrain/H_IFNgInd1 and M_IFNgTrain/M_IFNgInd1 for the human and mouse hosts, respectively. To further assess model robustness and generalization, a second independent test set (Independent Data set 2) was constructed for both human (H_IFNgInd2) and mouse (M_IFNgInd2) hosts. This data set reused the IFN-gamma-inducing peptides from Independent Data set 1 while incorporating new noninducing peptides from the unused portion of IEDB, ensuring no overlap with the Training or Independent Data set 1 sets.^15^

In addition to the data sets used for model training and evaluation, a separate collection of recently reported IFN-gamm-inducing and noninducing peptides was compiled from the Immune Epitope Database (IEDB) to construct a custom knowledge graph (IFNgKG). IFN-gamma-inducing peptides were selected from T-cell assays reporting IFN-gamma cytokine release, while noninducing peptides were collected from T-cell assays reporting cytokine responses excluding IFN-gamma or explicitly negative IFN-gamma results. To prevent redundancy and ensure data set independence, all peptides from the training and independent test sets (both positive and negative) were combined with the newly collected IFNgKG sequences and clustered using CD-HIT at a 40% sequence identity threshold. Only representative sequences from each cluster were retained, guaranteeing ≤40% similarity between IFNgKG and any sequences in the model development data sets. The final IFNgKG data set contained 169 IFN-gamma-inducing and 554 noninducing epitopes from human and mouse hosts. These sequences are entirely distinct from those used for training and testing, allowing the knowledge graph to contribute external, previously unseen information.

All peptide sequences in this study were standardized to a fixed length of 20 amino acids to ensure consistent embedding generation, and inference with the model. Shorter sequences were padded, and longer ones truncated to achieve uniform input dimensions. A summary of all data sets is provided in Table 1.

**Table 1:** Detailed Survey on IFNg Inducing Epitopes and Noninducing Epitopes

Host	Set	Inducing	Noninducing
Human	H_IFNgTrain	20394	20394
	H_IFNgInd1	5098	5098
	H_IFNgInd2	5098	33946
Mouse	M_IFNgTrain	6387	6387
	M_IFNgInd1	1596	1596
	M_IFNgInd2	1596	8139
Both	IFNgKG	169	554

### 2.2 Pretrained Language Model for Sequence Embedding

In the first stage of our workflow, each peptide sequence is transformed into a high-dimensional numerical representation using ESM2 ^23^ (Model: esm2_t33_650M_UR50D), a state-of-the-art protein language model from the Evolutionary Scale Modeling (ESM) family. ESM2 was selected for its superior performance in capturing long-range structural and functional signals in protein sequences, as demonstrated across diverse benchmarks such as remote homology detection, secondary structure prediction, and functional classification.

The ESM2 model was trained on the UR50/D 2021_04 data set, a large-scale, redundancy-reduced version of UniRef50 that includes millions of protein sequences, allowing the model to capture a wide spectrum of evolutionary and functional relationships. The ESM2 (650M) variant comprises 33 transformer layers and approximately 650 million parameters, enabling it to learn rich contextual embeddings (1280 dimensions per amino acid) that represent both local sequence motifs and long-range dependencies within peptides/proteins. These embeddings encode evolutionary, structural, and physicochemical patterns through self-supervised pretraining on masked language modeling.

In this study, ESM2 embeddings serve as the input representation for downstream integration with the knowledge graph in our IFNg_DeepKG framework. This combination of evolutionary signal and biological context enables robust prediction of IFN-gamma induction potential. For detailed architecture, training objectives, and comparative benchmarks, readers are referred to the original ESM2 publication.^23^

### 2.3 Knowledge Graph Retrieval-Augmented Generation

To enhance the predictive capacity of our model, we integrated retrieval-augmented generation (RAG) with a biologically informed knowledge graph (KG) constructed in Neo4j.^25^ Neo4j is one of the most widely adopted graph databases, designed to efficiently model and query highly connected data. Unlike traditional relational databases, Neo4j represents information as nodes, relationships, and properties, making it intuitive for capturing complex real-world networks such as biological pathways, social networks, and knowledge graphs. Its powerful query language, Cypher, allows for expressive and efficient traversal of relationships, which is particularly beneficial when working with multirelational data. Moreover, Neo4j provides strong scalability, integration with machine learning pipelines, and visualization tools, enabling scientists to uncover hidden patterns. Choosing Neo4j can therefore simplify handling complex relationships, improve query performance on connected data, and enhance interpretability through graph-based insights.

Traditional approaches to epitope classification, such as those relying solely on hand-crafted features (e.g., amino acid composition (AAC) and dipeptide composition (DPC)) or even protein language model (PLM) embeddings, typically treat each peptide in isolation. While these representations capture sequence-level features to some extent, they often fail to encode the biological context in which epitopes function, such as their source protein, host, or immunological relevance. Models built with hand-crafted features may encounter issues, such as feature redundancy, low feature correlation, and high feature dimensionality, which will lead to reduced accuracy in model recognition. This lack of contextual awareness may limit the generalizability of the resulting classifiers.^26–30^

In contrast, our framework explicitly encodes this biological context by leveraging a KG designed to represent the complex relationships surrounding epitopes. The KG consists of multiple biologically meaningful node types, including *Epitope* (annotated with attributes such as ID, sequence, and host), *Molecule*, *Organism*, and *Molecule Parent*. These nodes are connected via relationships (edges) such as *DERIVED_FROM*, *ORIGINATES_FROM, IS_VARIANT_OF*, and *BELONGS_TO*, which are sourced from IEDB metadata and UniProt annotations. These nodes and edges form triples in our KG space. By integrating this knowledge into the embedding search process, our method moves beyond sole sequence similarity to incorporate functional and biological similarity.

#### 2.3.1 Computation of Contextual Weights

The core challenge in integrating sequence-based retrieval (RAG) and knowledge graph (KG) data lies in balancing sequence distance with contextual relevance. Our framework addresses this by applying a novel weighted averaging scheme to retrieved entities.

The first step in the RAG+KG fusion process is the embedding retrieval of the top-5 closest matches from the database. This is performed by calculating the Euclidean distance (or L2 norm) between the input query embedding and all precomputed epitope embeddings in the database. The L2 distance, *d_i_*, is computed as the square root of the sum of the squared differences:

1 $$d_{i}=\sqrt{\overset{n}{\underset{j=1}{\sum }}{\left(q_{j}-e_{ij}\right)}^{2}}$$

Here, *q_j_* is the *j*-th element of the query embedding, *e_ij_* is the *j*-th element of the epitope embedding *e_i_* and *n* is the dimension of vectors *e_i_*, *q_j_*. We then sorts these distances and selects the indices of 5 epitopes exhibiting the minimum L2 distance.

Next, the method computes a context-dependent weight (*w*_*i*_) for each epitope embedding, resulting in a single, combined embedding (*W*_average_):

2 $$W_{\mathrm{average}}=\overset{5}{\underset{i=1}{\sum }}w_{i}\times e_{i}$$

where *e*_*i*_ is one of the top 5 embeddings, *w_i_* is its context-dependent weight.

The weights *w_i_* are derived by querying the Neo4j KG for shared biological relationships among the top-5 retrieved epitopes. Their retrieved IDs serve as a bridge between the vector space and the structured metadata space. In this approach, we assign a higher weight to epitopes that share more properties with their neighbors, indicating greater contextual similarity within the retrieval set. The computation proceeds in 3 steps:

1. Initialization and property retrieval: Each of the top-5 retrieved epitopes starts with an initial score **S** of 1.0. The system queries the Neo4j KG for four specific properties of each epitope: host, source molecule, molecule parent, and source organism.
2. Pairwise comparison and score accumulation: For each retrieved epitope pair *E_i_,E*_*j*_ (*i,j* ∈ {1,...,5},*i* ≠ *j*) , a pairwise comparison is performed to evaluate shared biological relationships for all categories. When two epitopes share a relationship within a specific category, a predefined incremental value is added to the accumulated scores of both epitopes. These values are defined in the empirically derived Relationship Map **M**, which assigns distinct importance levels to different biological relationship types. Following an ablation study, we determined the optimal Relationship Maps as *M*_human_ = {host:1.0, source_–_molecule:0.5,source_–_organism:0.6, molecule_–_parent:0.4}­for the H_IFNgTrain data set, and *M*_human_ = {host:1.0, source:0.6, source_–_organism:0.5, molecule_–_parent:0.4} for the M_IFNgTrain data set. For example, if epitopes *E*_1_ and *E*_2_ share the same host and source molecule under *M*_human_, values of 1.0 and 0.5 are added to both accumulated scores *S*(*E*_1_) and *S*(*E*_2_). This frequency-based scoring approach, where weights are derived from counting co-occurrence features, is a common mechanism in Graph Neural Networks and RAG systems.^31–37^ The hyperparameter optimization results for *M*_human_and M_mouse_ are described in Section 3.4.1 and Section 3.4.2.
3. Score normalization: The final contextual weight *w_i_* is obtained by normalizing the accumulated score of each epitope by the total sum of all accumulated scores. If no KG properties are available for the retrieved epitopes, uniform weights (*w*_*i*_ = 0.2) are used for 5 epitopes.

#### 2.3.2 Relationship Weight Hyperparameters

The values in the Relationship Map **M** are tunable hyperparameters determined through an extensive ablation study (Section 3.3) to ensure the weight hierarchy reflects the actual predictive contribution (importance) of each biological relationship. This approach, where specific relationship types are weighted based on their importance, is encouraged by established practices in Knowledge Graph Embedding (KGE) models.^38–41^ The results indicated that host consistently produced the highest predictive performance, while molecule parent contributed the least. Source molecule and source organism exhibited intermediate and comparable effects based on the main performance metrics. To encode this hierarchy, the incremental value for the host relationship was anchored at 1.0, while less frequently observed relationships between epitope pairs were assigned progressively smaller counting values. This strategy was empirically tested to mitigate their negative influence on overall performance. Experimental evaluations show that this configuration outperformed the uniform weighting strategy, where all relationship properties are regarded as evenly important. Overall, the Relationship Maps can capture the relative importance of biological relationships within the knowledge graph space, thereby enhancing the construction of weighted RAG embeddings.

#### 2.3.3 Final Fused Embedding

The final representation is obtained by a convex combination of the query embedding and the knowledge-informed embedding, adapting the ratio 9:1 previously optimized in our previous study.^42^ This weighting preserves the dominant signal from the PLM embedding while incorporating context information from the RAG+KG retrieval. Comparative experiments with other ratios such as 2:1 and 1:1, where knowledge-informed embeddings receive more weights, observe overfitting on independent test set (Supporting Information, Figure S6).

The benefits of this approach are multifold. Unlike feature engineering-based descriptors (AAC, DPC), which are limited in their capacity to capture higher-order relationships, and unlike sole PLM embeddings, which primarily encode sequence patterns, our RAG+KG strategy enables context-aware retrieval. When a knowledge graph lacks weight information, all facts are treated equally, assuming they are equally valid. This can cause misleading prioritizations. For instance, “Apples are red fruits” might outweigh “Apples are fruits” due to its specificity. Introducing weights, however, helps quantify factual reliability and corrects such oversimplified assumptions.

By grounding sequence embeddings in a graph of biological relationships, we effectively bias the search toward epitopes that are not only structurally similar but also functionally relevant in terms of host specificity and immunogenic potential. This results in improved performance in distinguishing IFN-gamma-inducing epitopes from non-IFN-gamma-inducing counterparts.

Figure 3 illustrates our implementation for generating fused embeddings between the queries and RAG+KG database.

**Figure 3:**
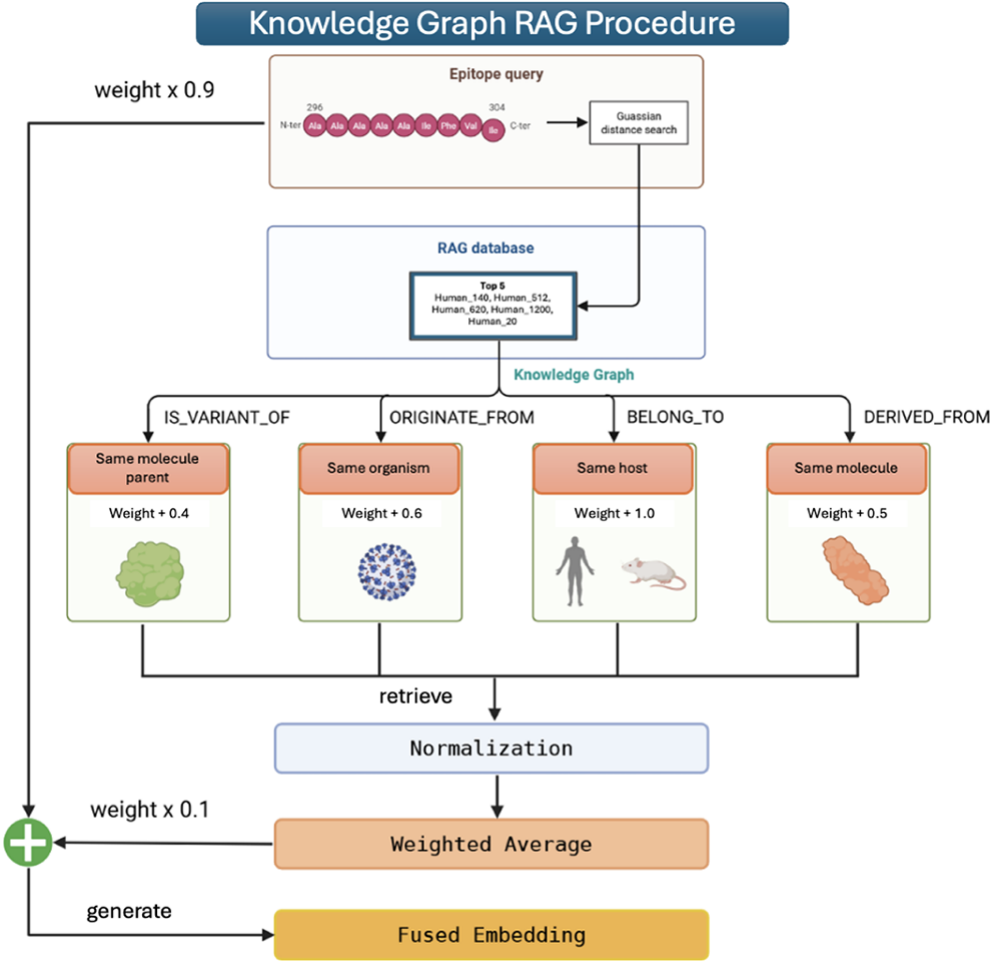
Knowledge Graph RAG mechanism to select top-5 relevant knowledge-enhanced epitope embeddings.

### 2.4 Multi-Scale Convolutional Neural Network (MSCNN)

The final component of the IFNg_DeepKG model is a Multi-Scale Convolutional Neural Network (MSCNN) that processes the fused embeddings to produce the final classification. This architecture is particularly well-suited for modeling biological sequences, as it employs parallel convolutional branches with different window sizes to capture motifs of varying lengths. This multiscale approach allows the model to learn both fine-grained, short-range patterns and broader, long-range contextual features simultaneously, which is essential for understanding the complex nature of protein sequences.

A key technical element of the MSCNN architecture is the use of separable convolution layers.^43^ Separable convolution layers are a powerful optimization technique that decomposes the standard convolution operation into two distinct, more computationally efficient steps: a depth-wise convolution and a point-wise convolution. The depth-wise convolution applies a single filter to each input channel, while the point-wise convolution uses a 1 × 1 filter to combine the outputs from the depth-wise step. This decomposition significantly reduces the number of parameters and computational complexity, which helps to mitigate overfitting and allows for the development of deeper and more efficient networks.

The depthwise convolution for a layer with a window size w is

3 $$\mathrm{Depthwise}\left(x\right)=\overset{w-1}{\underset{i=0}{\sum }}K_{i}.x_{:,:,i}$$

where *K_i_* is the kernel for position *i*. The pointwise convolution follows:

4 Pointwise(x)=W.x

where *W* is a 1 × 1 kernel mixing channels. These layers are followed by max-pooling, flattening, dropout, and dense layers for classification. This multiscale design allows MSCNN to detect local motifs (small windows) and broader patterns (larger windows), crucial for epitope identification.

### 2.5 Performance Evaluation

We evaluated the effectiveness of our model through a comprehensive set of well-established performance indicators. These include Sensitivity, Specificity, Accuracy, Matthews Correlation Coefficient (MCC), Precision, F1 score, and the Area Under the Receiver Operating Characteristic Curve (AUC). Sensitivity (or Recall) measures the proportion of correctly identified positive cases, while Specificity quantifies the proportion of negatives that are accurately recognized. Accuracy reflects the overall rate of correct predictions. MCC offers a single balanced score by incorporating all four elements of the confusion matrix, which is especially beneficial when dealing with uneven class distributions. Precision indicates the fraction of predicted positives that are truly positive, and the F1 score provides a harmonic balance between Precision and Recall, making it valuable in scenarios with class imbalance. Lastly, AUC captures the model’s ability to separate positive from negative samples over different decision thresholds. The mathematical formulations of these metrics are presented as follows:

5 $$\mathrm{Sensitivity}=\left(\frac{\mathrm{TP}}{\mathrm{TP}+\mathrm{FN}}\right)$$

6 $$\mathrm{Specificity}=\left(\frac{\mathrm{TN}}{\mathrm{TN}+\mathrm{FP}}\right)$$

7 $$\mathrm{Accuracy}=\left(\frac{\mathrm{TP}+\mathrm{TN}}{\mathrm{TN}+\mathrm{FP}+\mathrm{TP}+\mathrm{FN}}\right)$$

$$\mathrm{MCC}=\frac{\left(\mathrm{TP}\times \mathrm{TN}\right)-\left(\mathrm{FP}\times \mathrm{FN}\right)}{\sqrt{\left(\mathrm{TP}+\mathrm{FP}\right)\left(\mathrm{TP}+\mathrm{FN}\right)\left(\mathrm{TN}+\mathrm{FP}\right)\left(\mathrm{TN}+\mathrm{FN}\right)}}$$ 8

9 $$\mathrm{Precision}=\left(\frac{\mathrm{TP}}{\mathrm{TP}+\mathrm{FP}}\right)$$

10 $$F1=\left(2\times \frac{\mathrm{precision}\times \mathrm{recall}}{\mathrm{precision}+\mathrm{recall}}\right)$$

where TP denotes True Positive, TN denotes True Negative, FP denotes False Positive, and FN denotes False Negative. Besides, the AUC values will be visually represented through the ROC graphs.

### 2.6 Deep Learning Architecture

The proposed model is based on a Multi-Scale Convolutional Neural Network (MSCNN) designed to capture both local and global sequence patterns relevant to IFN-gamma-inducing epitope prediction. The overall architecture is summarized in Table 2. The MSCNN consists of multiple convolutional branches with varying kernel sizes to extract multiscale contextual representations from the input embeddings. These extracted features are concatenated and passed through fully connected layers for classification.

**Table 2:** MSCNN Architecture Summary^a^

Layer	Type	Kernel size	Filter	Output shape	Parameters
Input	-	-	-	(batch, 1, 20, 1280)	0
Conv2Dx4	SeparableConv2D	(1,2), (1,4), (1,6), (1,8)	1024	(batch, 1, 19, 1024) (batch, 1, 17, 1024) (batch, 1, 15, 1024) (batch, 1, 13, 1024)	∼5.27M
MaxPool × 4	MaxPooling2D	(1,19), (1,17), (1,15), (1,13)	-	(batch, 1, 1, 1024) × 4	0
Concat	-	-	-	(batch, 4096)	0
Dropout	Dropout (0.7)	-	-	(batch, 4096)	0
Dense	Dense	-	500	(batch, 500)	∼2.05M
Output	Dense	-	2	(batch, 2)	1,002
Total trainable parameters					∼7.32M

a In Mouse training set, window (1,8) is dismissed from combination, so total learnable parameters are smaller.

To ensure robust and fair optimization, the model’s hyperparameters were carefully selected through systematic tuning and ablation analyses. The detailed training configuration, including hyperparameters and computational hardware, is listed in Table 3. The default learning rate of 1 × 10^– 3^ was validated via 5-fold cross-validations (Table S5).

**Table 3:** Training Hyperparameters and Hardware

Hyperparameter	Value
Optimizer	Adam
Learning rate	1 × 10^–3^
Batch size	256
Filter size	1024
Hidden layer	500
Epoch	20
Loss	Categorical cross-entropy
Regularization	L2 (1*e* ^–3^), Dropout (0.7)
Hardware	NVIDIA GeForce RTX 3090 Ti, CUDA 12.6, Tensorflow 2.15.0

Batch size was optimized through an ablation study (Table S6), and a batch size of 256 yielded the best generalization across both training data sets. Other architectural parameters, such as the combination of multiple convolutional windows, number of filters, and number of hidden layers, were systematically analyzed and reported in Tables S2–S4, which present the 5-fold cross-validation results for each configuration.

This comprehensive hyperparameter optimization process ensures that the final MSCNN architecture provides both stable and high-performing representations across species-specific data sets, facilitating robust prediction of IFN-gamma-inducing peptides.

## 3 Results and Discussion

### 3.1 Performance of Model Hyperparameters

A comprehensive hyperparameter search was conducted to optimize the performance of the MSCNN model. Table S1, located in the Supporting Information, details the performance of the model using various single window sizes. The results for the human data set (H_IFNgTrain) indicate that a window size of 8 achieved the highest accuracy (0.7965), MCC (0.5944), and AUC (0.8703), while for the mouse data set (M_IFNgTrain), a window size of 6 yielded the best performance in these metrics. The observation that no single window size is universally optimal across different host data sets validates the fundamental rationale for a multiscale approach.

The superiority of the multiscale approach is further confirmed by the results in Table S2. Combining multiple windows consistently improved performance. For the human training data set, the combination of windows {2, 4, 6, 8} resulted in the highest accuracy (0.8136), MCC (0.6288), and AUC (0.8854). Similarly, for the mouse data set, the combination of windows {2, 4, 6} achieved the highest accuracy (0.7500), MCC (0.5026) and AUC (0.8196), while {2, 4, 6, 8} had the highest AUC (0.8199). These findings justify the selection of the multiwindow MSCNN architecture as the final classifier. Additional analyses on filter and hidden layer parameters, presented in Tables S3 and S4, show that 1024 filters plus 500 hidden layer nodes were optimal for human and mouse data sets, respectively, demonstrating a robust and data-driven optimization process.

To ensure optimal predictive performance, we subjected key training hyperparameters to an extensive ablation analysis. The impact of the learning rates including 1*e* ^– 2^, 1*e* ^– 3^, 1*e* ^– 4^, 3*e* ^– 4^, 5*e* ^– 4^ were systematically tested to determine the ideal value for the Adam optimizer. As demonstrated by the performance metrics presented in Figure S5, a learning rate of 1*e* ^–3^ provided the superior performance, achieving an AUC of 0.8889 and a MCC of 0.6414 on the H_IFNgTrain data set. For the M_IFNgTrain set, this rate similarly led to the best results with an AUC of 0.8314 and an MCC of 0.5192.

We also optimized the batch size to balance computational efficiency with the stability of the loss gradient. The results of the 5-fold cross-validation, detailed in Figure S6, showed that a batch size of 256 consistently yielded the highest predictive performance metrics. This larger batch size was adopted to provide a more reliable estimate of the gradient, thus promoting better convergence and generalization across both human and murine training data sets.

### 3.2 Performance of Different Embedding Features

To quantify the value of using a modern protein language model, a comparison was performed between ESM2 features and traditional feature representations on the two training data sets (Figure S1). The results demonstrate that the ESM2 model dramatically outperforms traditional features such as AAC, DPC, and One-hot encoding across all performance metrics, including AUC, Accuracy, and MCC. For instance, on the H_IFNgTrain data set, ESM2 achieved an AUC of 0.8889, significantly higher than the 0.6986 of AAC or 0.7372 of DPC. This finding confirms that ESM2’s contextual embeddings capture far more biologically relevant information than simple compositional or positional features.

A further comparison was conducted to evaluate ESM2 against other leading protein language models such as ProtT5 ^22^ and TAPE^44^ (Figure S2). On the human data set H_IFNgTrain, ProtT5, ESM2, and TAPE demonstrated very competitive performance, with AUCs of 0.8810, 0.8889, and 0.8779, respectively. On the mouse data set M_IFNgTrain, ProtT5 and TAPE were nearly identical in performance, with AUCs of 0.8059 and 0.8045, while ESM2 outweighed them with AUC of 0.8314. The radar plots in Figure S2 visually show that while all three PLMs perform well, ESM2 consistently holds a slight edge or is highly competitive across a range of evaluation metrics, justifying its selection as the base model for this study.

Compared to earlier protein language models such as TAPE (768 dimensions) and ProtTrans (1024 dimensions), ESM2 (1280 dimensions) exhibits markedly superior representation capacity and generalization ability. This improvement stems from its deeper transformer architecture, expanded pretraining corpus, higher-dimensional embeddings, and a refined masked language modeling objective that enables more effective capture of complex sequence semantics and structural dependencies. These advantages make ESM2 particularly effective for downstream bioinformatics tasks, including structure prediction, function classification, and residue-level property inference, even in cases with limited labeled data.

### 3.3 Performance of Various Machine Learning Models

To evaluate the effectiveness of the proposed Multi-Scale Convolutional Neural Network (MSCNN), we compared its performance with a range of traditional and deep learning models using ESM2 embeddings as input features (Figure S3). The benchmarked models included Convolutional Neural Network (CNN), Multilayer Perceptron (MLP), Support Vector Classifier (SVC), K-Nearest Neighbors (KNN), Random Forest (RF), Extra Trees (ET), Long Short-Term Memory (LSTM), Bidirectional LSTM (BiLSTM), Gated Recurrent Unit (GRU) and Bidirectional GRU (BiGRU).

Baseline classifiers were implemented with the following architectures and hyperparameters: CNN (two Conv2D layers with 32 and 64 filters, kernel size (1,3), max-pooling, 128-unit dense layer, 0.7 dropout), MLP (128, 64, and 2 dense layers), SVC (C = 0.001), KNN (*k* = 3), RF (10 trees), ET (100 trees), and recurrent models (LSTM/GRU/BiLSTM/BiGRU with 64 hidden units followed by a 32-unit dense layer), all trained for 20 epochs with batch size 64.

Across both the H_IFNgTrain and M_IFNgTrain data sets, MSCNN consistently achieved the highest performance across all key metrics AUC, F1-score, MCC, and Accuracy, demonstrating its superior ability to capture discriminative sequence representations. For the human data set (H_IFNgTrain), MSCNN achieved an AUC of 0.8889 and an F1-score of 0.8155, outperforming both the traditional CNN (AUC = 0.8201, F1 = 0.7425) and MLP (AUC = 0.8066, F1 = 0.7320). Similarly, for the mouse data set (M_IFNgTrain), MSCNN obtained the best results (AUC = 0.8314, F1 = 0.7555), exceeding those of CNN (AUC = 0.7323, F1 = 0.6702) and MLP (AUC = 0.7258, F1 = 0.6679). Other recurrent neural network architectures, including LSTM, BiLSTM, GRU, and BiGRU, achieved performance comparable to the SVC model (AUC = 0.8460 in Human Data set and 0.7515 in Mouse Data set), with average AUC values of approximately 0.8338 on the H_IFNgTrain and 0.7594 on the M_IFNgTrain. In contrast, traditional machine learning models such as KNN and RF exhibited notably lower predictive performance, with AUCs ranging from 0.7990–0.8093 for the H_IFNgTrain and 0.6741–0.6880 for the M_IFNgTrain.

The superior performance of MSCNN can be attributed to its multiscale convolutional architecture, which captures hierarchical contextual dependencies across multiple receptive fields, enabling it to learn both local residue-level patterns and global sequence-level representations. In contrast, traditional CNNs are limited to a single convolutional scale, while MLPs lack spatial awareness of sequential features. Furthermore, while recurrent models such as Bi-LSTM and Bi-GRU exhibit competitive performance, they require significantly higher computational cost and training time. Overall, MSCNN provides an optimal balance between predictive accuracy and efficiency, justifying its adoption as the backbone architecture for the IFNg_DeepKG framework.

### 3.4 The Impact of Knowledge Graph Integration

#### 3.4.1 Single Relationship Effect

We performed an ablation study to evaluate the individual contribution of each weighting component. Specifically, we trained the model using only one weight category at a time (i.e., Host only, Molecule only, Parent only, and Organism only) and compared the results to the model trained with all weights combined. The detailed results are presented in Figure S4, showing the performance on both H_IFNgTrain and M_IFNgTrain data sets.

As shown in Figure S4, each individual component contributes positively to model performance, but no single category alone achieves the same level of accuracy or robustness as the combination of all weights. The best individual performance for the Human data set is achieved using the Host weight (Accuracy = 0.9229, MCC = 0.8467, AUC = 0.9717, F1 = 0.9246), while for the Mouse data set, the Host weight again performs best (Accuracy = 0.8604, MCC = 0.7216, AUC = 0.9329, F1 = 0.8612). However, integrating all four components leads to further improvement in both data sets (Human: Accuracy = 0.9348, MCC = 0.8703, AUC = 0.9778, F1 = 0.9360; Mouse: Accuracy = 0.8631, MCC = 0.7263, AUC = 0.9324, F1 = 0.8629), demonstrating that the combination of all weight categories synergistically enhances model performance. The small standard deviation observed across values indicates that each weighting strategy exhibits high stability.

These results validate the importance of each term and confirm that their joint contribution is essential for achieving optimal predictive accuracy.

#### 3.4.2 Multiple Relationship Integration

Figure S5 illustrates the performance comparison of multiple weighting strategies applied to both H_IFNgTrain and M_IFNgTrain data sets. The evaluated strategies include: {“Uniform”: (1.0, 1.0, 1.0, 1.0)}, {“Set_A”: (1.0, 0.9, 0.7, 0.8)}, {“Set_B”: (1.0, 0.6, 0.4, 0.5)}, {“Set_C”: (1.0, 0.3, 0.1, 0.2)}, {“Set_D”: (1.0, 0.8, 0.7, 0.9)}, {“Set_E”: (1.0, 0.5, 0.4, 0.6)}, and {“Set_F”: (1.0, 0.2, 0.1, 0.3)}, where weights correspond to the attributes {“host”, “source_molecule”, “molecule_parent”, “source_organism”}. Across all configurations, the model consistently achieved high accuracy, MCC, AUC, and F1 scores, with only marginal fluctuations among different strategies. These results suggest that the model’s predictive capability remains stable and resilient under diverse weighting conditions, reflecting its robustness and balanced feature utilization.

For the H_IFNgTrain data set, the Set_E weighting strategy achieved the best overall performance, with an mean accuracy of 0.9403, MCC of 0.8812, AUC of 0.9815, and F1 score of 0.9414. This represents a modest yet consistent improvement over the uniform weighting baseline, suggesting that the weight adjustment in Set_E effectively enhances the model’s discriminative ability.

For the M_IFNgTrain data set, on the other hand, the Set_B weighting strategy yielded the highest performance, with a mean accuracy of 0.8673, MCC of 0.7353, AUC of 0.9375, and F1 score of 0.8670. These results indicate that Set_B provides the most balanced optimization between sensitivity and specificity for mouse data.

Collectively, these findings demonstrate that appropriate weighting can provide minor yet meaningful performance gains while maintaining high stability in both human and mouse data sets.

#### 3.4.3 Effect of IFN-Gamma-Inducing Status on Model Performance

A key element represented in the KG structure is the IFN-gamma-inducing status of each epitope. However, during deep learning model training, it is critical to exclude label information from the input data to prevent bias and avoid data leakage, which could otherwise artificially inflate model performance and compromise generalization.

To evaluate whether incorporating this status in the RAG embedding could potentially introduce data leakage, we conducted an comparative experiment, noting model performance with and without IFN-gamma associations during KG construction (Table S7). The results show that including the IFN-gamma-inducing relationship led to a slight improvement in predictive performance for both human (Acc: 0.9465 vs 0.9348; MCC: 0.8938 vs 0.8703; AUC: 0.9840 vs 0.9778) and mouse data sets (Acc: 0.8705 vs 0.8631; MCC: 0.7412 vs 0.7263; AUC: 0.9378 vs 0.9324). However, in the case of binary classification (IFN-gamma-inducing vs noninducing), the risk of information leakage increases as the RAG-KG database grows. This occurs because a query may inadvertently access the true labels of retrieved candidates, leading to biased predictions. To prevent such potential leakage and ensure fair evaluation, we intentionally excluded the IFN-gamma-inducing status from the weighting strategy.

#### 3.4.4 Effect of Fusion Ratios

We evaluated our model under various fusion ratios between the query and KG-enhanced context vectors such as 9:1, 2:1, and 1:1 across both H_IFNgTrain and M_IFNgTrain data sets to assess the impact of context integration on model performance with an imbalanced independent test.

During training, the 1:1 ratio achieved the highest overall metrics, indicating strong fitting capacity with excellent sensitivity, specificity, and AUC values in both human and mouse data sets. However, when evaluated on the imbalanced Independent Data set 2, the 1:1 and 2:1 ratios exhibited signs of overfitting, with noticeably reduced generalization performance. This phenomenon likely arises because the RAG+KG database remains relatively limited in size, while the number of queries is considerably larger. As a result, many biologically similar queries tend to retrieve overlapping top-5 epitope embeddings. When these shared embeddings receive disproportionately high weights, the model’s decision boundary may shift toward the weighted embeddings rather than the unique query representations, leading to reduced generalization on independent data sets.

In contrast, the 9:1 ratio maintained stable and balanced performance across all data sets, achieving strong predictive power in training while preserving robustness on the independent test set. This trend is clearly depicted in Figure S6.

#### 3.4.5 Performance on Two Independent Tests

Table S8 compares the predictive performance between the baseline ESM2 model and the Knowledge Graph RAG-enhanced variant (RAGKG-ESM2) across both training and independent test data sets. In the 5-fold cross-validation results, RAGKG-ESM2 consistently outperformed the standard ESM2 model in both H_IFNgTrain and M_IFNgTrain sets. Specifically, the integration of knowledge graph retrieval led to substantial gains in sensitivity (0.9524 vs 0.7948 in human and 0.8615 vs 0.7433 in mouse) and MCC (0.8703 vs 0.6414 in human and 0.7263 vs 0.5192 in mouse), indicating enhanced fitting ability and more balanced classification between IFN-gamma-inducing and noninducing peptides.

This improvement extended to the independent test data sets, demonstrating the strong generalization capacity of RAGKG-ESM2. In H_IFNgInd1 and M_IFNgInd1, the RAGKG-ESM2 variant achieved outstanding AUC values of 0.9878 and 0.9510, respectively, surpassing the ESM2 model by over 0.07 in both cases. Similarly, MCCs increased by more than 0.25, reflecting a more robust and reliable prediction across species. Notably, in the more challenging imbalanced independent test set (H_IFNgInd2 and M_IFNgInd2), RAGKG-ESM2 preserved high predictive power, with AUC improvements of 0.19 (human) and 0.26 (mouse) compared to ESM2.

Overall, these findings demonstrate that integrating the Knowledge Graph RAG module into ESM2 embeddings significantly enhances both learning and generalization performance. By enriching sequence representations with biologically relevant relational knowledge, RAGKG-ESM2 achieves more context-aware embedding construction and markedly superior prediction outcomes on both human and mouse data sets. Figure S7 presents ROC curves for both ESM2 and its RAGKG-enhanced variant.

### 3.5 A Visual Insight into Feature Learning

#### 3.5.1 t-SNE Visualization

The effectiveness of the MSCNN in learning a discriminative feature space is further illustrated by the t-SNE visualizations in Figure 4 and Figure S8.

**Figure 4:**
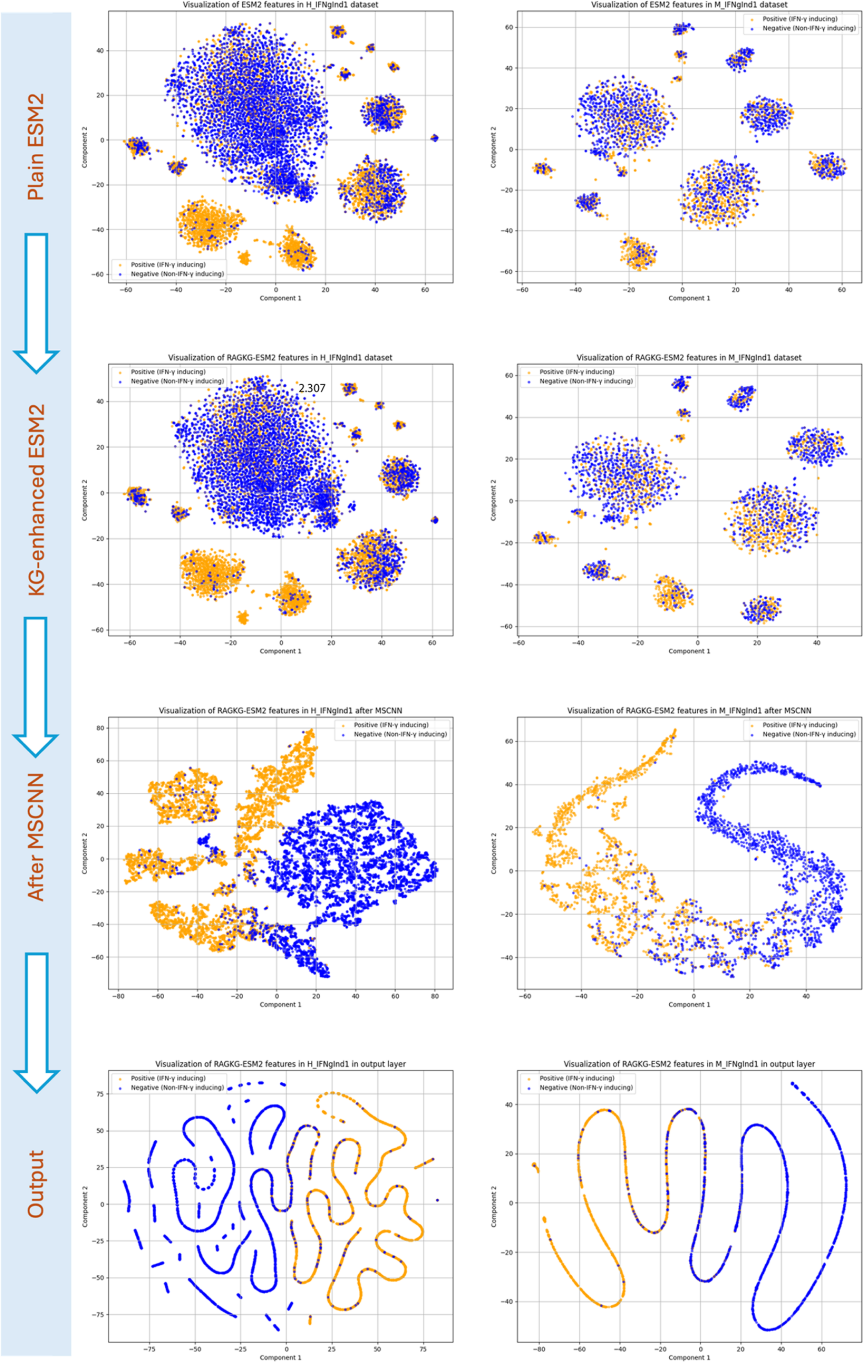
Visualization of independent test data sets (H_IFNgInd1 and M_IFNgInd1) using the MSCNN model. The left panels correspond to the human host data set (H_IFNgInd1), and the right panels correspond to the mouse host data set (M_IFNgInd1). Each data set is visualized across four key stages of the prediction pipeline: (1) ESM2 embeddings before integration with the RAG–KG module, (2) embeddings during the RAG–KG fusion process, (3) feature representations after processing through the MSCNN model, and (4) the final output layer. These visualizations highlight how the data distributions evolve and become more discriminative as they progress through each stage of the model.

The visualization in Figure 4 depicted the distribution of the ESM2+RAG-KG-enhanced embeddings before and after being processed by the MSCNN model for the Independent Data sets 1. Before applying the MSCNN, the embeddings of IFN-gamma-inducing and noninducing epitopes appeared partially intermingled, showing no clear cluster boundaries between the two classes. After integrating the RAG-KG embeddings, the representations became more structured, with positive and negative samples forming more compact and distinct groups, although some degree of overlap still remained. However, after being passed through the MSCNN’s feature learning layers, the model’s output representations show a much clearer separation of the two classes.

The t-SNE visualizations for the imbalanced H_IFNgInd2 and M_IFNgInd2 data sets (Figure S8) similarly provide compelling visual evidence that the MSCNN is successfully transforming the initial embeddings into a more biologically meaningful, discriminative space, which directly explains the model’s high classification performance.

#### 3.5.2 GRAD-CAM Visualization

To further interpret the decision mechanism of the MSCNN model, we performed GRAD-CAM visualization on representative cases of true positive (TP), false positive (FP), true negative (TN), and false negative (FN) predictions (Figures S9–S10). Overall, our model can learn the host-specific residue composition and positional preferences.^15^

In the H_IFNgInd1 data set, with TP example (P_te_Seq_2: LPRQRAYL), the model accurately recognized the IFN-gamma-inducing peptide by assigning strong activation (>0.95) to the “LP” residues, which correspond to the most discriminative motif in human epitopes, while correctly down-weighting noninformative residues such as R and A. In contrast, the FP case (N_te_Seq_3506: MIEEIDADGSGTVDF) revealed enriched activations on I, S, and T residues, which frequently associated with IFN-gamma induction. This suggests the model captured biologically plausible but experimentally misclassified patterns. The TN peptide (N_te_Seq_4895: VPAKSVCGPVYCFTP) showed minimal activation (<0.05) across all positions, indicating the model’s confidence in identifying the absence of Th1-associated motifs. Finally, the FN case (P_te_Seq_18: HEIHIGYL) displayed high attention to residue H, E at position 2, 3 but overlooked critical C-terminal L residue (depleted residue toward IFN-gamma-inducing epitopes), leading to a missed positive prediction.

Similarly, GRAD-CAM visualization on the M_IFNgInd confirmed that the MSCNN model captures meaningful residue-level features, though the classification boundary is more intricate than in the human data set due to mixed enrichment and depletion patterns in the mouse host. In the TP case (P_test_1: VSVVFAAL), the model correctly identified an IFN-gamma-inducing peptide, highlighting residues V and A as key contributors, though moderate activation on the depleted residue L (∼0.6) suggests slight ambiguity. The FP example (N_test_522: KQTCNSSAV) showed misclassification caused by elevated activation on T and V despite great activation (>0.65) on depleted residues S, reflecting conflicting sequence cues. The TN peptide (N_test_2010: TVVNKVLIPM) exhibited near-zero activation across most residues, indicating correct rejection of a noninducing sequence despite a minor hotspot at position 1 (T). Conversely, the FN case (P_test_3: LYQLENYC) was missed because the model gave remarkable attention (>0.6) on the N-terminal N residue together with the critical C-terminal residues. This confusion may have influenced the decision. These results suggest that, while the model learns biologically interpretable motifs, the heterogeneous residue composition in the mouse host leads to a more complex decision landscape, explaining the relatively lower predictive performance compared to the human data set.

Collectively, these visualizations demonstrate that MSCNN learns interpretable and biologically grounded residue-level patterns, even when occasional errors arise from subtle motif overlaps or experimental noise.

#### 3.5.3 SHAP Visualization

To further interpret the decision behavior of our model, we applied SHAP (SHapley Additive exPlanations) analysis to identify the most influential embedding dimensions contributing to the classification of IFN-gamm-inducing epitopes across both Human and Mouse data sets (Figure S11–S12). Each SHAP summary plot illustrates the top-10 most important features, where each dot represents an individual sample, and the *x*-axis reflects the SHAP value, the degree to which a feature drives the prediction toward either Class 0 (Noninducing) or Class 1 (IFN-gamma-Inducing). Red denotes high feature values, and blue indicates low feature values.

Across all four data sets (H_IFNgInd1, H_IFNgInd2, M_IFNgInd1, and M_IFNgInd2), the embedding dimensions Dim_1160 and Dim_234 consistently emerged as the most dominant predictors, demonstrating their universal significance in determining IFN-gamma induction potential. In the Human data sets, Dim_234 was consistently critical in both panels, while high Dim_1160 strongly contributed to IFN-gamma-inducing predictions in H_IFNgInd1. Conversely, in H_IFNgInd2, high Dim_234 and high Dim_89 served as antagonistic predictors, with the former favoring inducing epitopes and the latter favoring noninducing ones. Similarly, in the Mouse data sets, Dim_1160 and Dim_234 again dominated as the most informative dimensions across both cohorts, while low Dim_1210 in M_IFNgInd2 uniquely appeared as a strong positive determinant of IFN-gamma induction.

Overall, the SHAP visualization highlights that a core subset of embedding dimensions, particularly Dim_1160 and Dim_234, encode generalizable biological information that governs IFN-gamma-inducing potential across species. Meanwhile, certain data set-specific dimensions, such as Dim_89 and Dim_1210, capture host-dependent variations, reflecting how species-specific immunological patterns subtly shape the model’s interpretation of sequence-level features.

### 3.6 Comparison with Existing Methods

To assess the generalizability and competitiveness of the proposed IFNg_DeepKG framework, we compared its performance with several existing state-of-the-art models, including TransPHLA,^19^ CapHLA,^20^ BigMHC,^21^ and IFNepitope2,^15^ using two independent test sets for both human and mouse data sets (Table 4). These benchmark models represent diverse methodological paradigms such as Transformer-based encoders (TransPHLA, BigMHC), attention-guided networks (CapHLA), and machine learning ensemble classifiers (IFNepitope2). For fair comparison, we utilized their networks for embedding generation from our data sets.

**Table 4:** Comparison of Model Performance between Our Model and Other Existing Methods

Set	Models	Sens	Spec	Acc	MCC	AUC	F1	Pre
Independent Test 1								
H_IFNgInd1	**Ours (2025)**	97.80	94.64	96.22	0.92	0.99	0.96	0.95
	IFNepitope2 (2024)^a^ ^15^	83.31	82.88	83.09	0.66	0.90	0.83	-
	TransPHLA (2022)^19^	85.97	43.47	64.72	0.33	0.76	0.71	0.60
	CapHLA (2025)^20^	69.05	85.96	77.50	0.56	0.84	0.75	0.83
	BigMHC (2023)^21^	70.50	81.37	75.93	0.52	0.84	0.75	0.79
M_IFNgInd1	**Ours (2025)**	85.28	91.42	88.35	0.77	0.95	0.88	0.91
	IFNepitope2 (2024)^15^	76.75	77.82	77.29	0.55	0.85	0.77	-
	TransPHLA (2022)^19^	82.89	28.38	55.64	0.13	0.56	0.65	0.54
	CapHLA (2025)^20^	79.76	55.83	67.79	0.37	0.76	0.71	0.64
	BigMHC (2023)^21^	63.66	61.59	62.63	0.25	0.67	0.63	0.62
Independent Test 2								
H_IFNgInd2	**Ours (2025)**	93.92	83.71	85.04	0.59	0.94	0.62	0.46
	IFNepitope2 (2024)^15^	83.31	80.76	81.85	0.64	0.88	0.80	-
	TransPHLA (2022)^19^	63.87	23.65	28.90	–0.10	0.35	0.19	0.11
	CapHLA (2025)^20^	75.05	57.09	59.43	0.22	0.72	0.33	0.21
	BigMHC (2023)^21^	69.67	58.88	60.29	0.19	0.70	0.31	0.20
M_IFNgInd2	**Ours (2025)**	88.47	83.62	84.42	0.59	0.93	0.65	0.51
	IFNepitope2 (2024)^15^	76.77	75.92	76.06	0.42	0.82	0.51	-
	TransPHLA (2022)^19^	97.87	3.74	19.17	0.03	0.76	0.28	0.17
	CapHLA (2025)^20^	69.61	50.77	53.86	0.15	0.65	0.33	0.22
	BigMHC (2023)^21^	66.67	40.40	44.70	0.05	0.56	0.28	0.18

a Results are collected from Supplementary Table S6, S7.^15^ Authors did not provide Precision metrics on Independent Data set 2.

Across all independent tests, our model consistently achieved the highest overall accuracy, MCC, and AUC, confirming its superior predictive robustness. For the human data set (H_IFNgInd1), IFNg_DeepKG achieved an AUC of 0.99, accuracy of 96.22%, and MCC of 0.92, outperforming IFNepitope2 (AUC = 0.90, Acc = 83.09%, MCC = 0.66), TransPHLA (AUC = 0.76, Acc = 64.72%), CapHLA (AUC = 0.84, Acc = 77.50%), and BigMHC (AUC = 0.84, Acc = 75.93%). Similarly, for the mouse data set (M_IFNgInd1), our model obtained an AUC of 0.95 and MCC of 0.77, exceeding those of IFNepitope2 (AUC = 0.85, MCC = 0.55) and the Transformer-based models, which showed poorer generalization, particularly TransPHLA (AUC = 0.56, MCC = 0.13).

On the more challenging Independent Test 2, which includes previously unseen sequences, our model maintained superior robustness, achieving AUC = 0.94 (H_IFNgInd2) and AUC = 0.93 (M_IFNgInd2), while other models experienced significant performance drops. Notably, TransPHLA and CapHLA exhibited unstable behavior, with sensitivity–specificity imbalance and lower MCC values (e.g., TransPHLA, MCC = −0.10 in H_IFNgInd2 and 0.03 in M_IFNgInd2). In contrast, our model preserved balanced sensitivity and specificity across data sets (93.92% and 83.71% for H_IFNgInd2, 88.47% and 83.62% for M_IFNgInd2, respectively).

The strong generalization ability of IFNg_DeepKG can be attributed to its RAG-Knowledge Graph fusion strategy, which enhances biological interpretability and mitigates overfitting, and its multiscale CNN backbone, which effectively captures both local and global sequence dependencies. Furthermore, our approach (∼7.32 M parameters) demonstrates better computational efficiency compared to Transformer-based models such as BigMHC (∼36.8 M parameters) and CapHLA (∼21 M parameters), while slightly larger but substantially more expressive than TransPHLA (∼1.5 M parameters) using partly Transformer-based encoders.

Overall, these results confirm that IFNg_DeepKG surpasses existing methods in predictive performance, robustness across species, and computational efficiency, establishing it as a reliable and biologically interpretable tool for IFN-gamma-inducing epitope prediction.

### 3.7 Case Studies in Precision Medicine

To demonstrate the model’s practical utility, a case study was performed on five specific epitopes (Table S9), all of which were correctly predicted as IFN-gamma-inducing.

Epitope ID 103041 and Epitope ID 104630 (diabetes-related): These epitopes, correctly predicted to be IFN-gamma-inducing, are relevant in the context of Type 1 Diabetes (T1D).^45^ T1D is an autoimmune disease where T-cell responses against self-antigens are a critical pathogenic factor. The ability to identify such epitopes is fundamental to understanding the immune-mediated destruction of pancreatic beta cells in T1D and could be instrumental in designing antigen-specific immunotherapies that aim to tolerize the immune system and halt the disease process.

Epitope ID 102639 (COVID-19 related): Correctly identified as an IFN-gamma-inducing epitope, this peptide from the SARS-CoV-2 ^46^ spike protein is of great clinical significance. T-cell responses to the SARS-CoV-2 spike protein are known to be critical for viral clearance and long-term immunity. This specific epitope is recognized for its ability to elicit a strong IFN-gammaresponse in human peripheral blood mononuclear cells, and it is a key target for next-generation vaccines aimed at achieving broad and robust immunity against various SARS-CoV-2 variants of concern.

Epitope ID 102926 (COVID-19 related): Similarly, this epitope, correctly predicted by the model, is involved in the T-cell-mediated immune response to COVID-19. The identification and characterization of such nonspike epitopes are vital for developing universal vaccine candidates that offer protection beyond the rapidly mutating spike protein. The ability of our model to accurately predict this epitope’s function confirms its utility in scrutinizing and predicting T-cell immunogenicity hotspots across the entire viral proteome.

Epitope ID 7493 (Alzheimer’s disease-related): The model’s correct prediction of this epitope, associated with Alzheimer’s disease, highlights its potential in fields beyond infectious diseases and cancer. Alzheimer’s disease is characterized by the progressive accumulation of amyloid-beta (Aβ) and Tau protein aggregates.^47^ Identifying epitopes on these pathogenic proteins is crucial for the development of targeted immunotherapies, such as therapeutic antibodies (e.g., Aducanumab, Lecanemab, Donanemab) that aim to clear these aggregates from the brain. The ability to accurately predict such epitopes is a significant step toward rational drug design for neurodegenerative diseases.

Finally, we explored the retrieved epitopes within the Neo4j knowledge graph to visualize their semantic and biological relationships with the query sequence human_7493 (Figure S13). The resulting graph-based map reveals how our Knowledge Graph (KG) features enhance interpretability by elucidating the relevance between the query and its top retrieved candidates. This relational insight provides a deeper understanding of the underlying biological connections, enabling researchers to identify functionally similar epitopes and potentially design novel peptide therapeutics with comparable immunological activity.

### 3.8 Therapeutic Implications and Research Limitations

The high predictive performance of IFNg_DeepKG, evidenced by an AUC of 0.99, suggests its substantial therapeutic potential. The model can reliably guide the design of peptide-based vaccines and immunotherapies for infectious diseases and cancer by identifying the most potent and relevant epitopes. The model’s reliance on a knowledge graph further enhances its utility by allowing it to learn disease-specific relationships and identify conserved motifs, which is crucial for diseases with complex immune interactions. This capability enables a more targeted and effective approach to therapeutic design.

Despite its success, this research has several inherent limitations. The model’s performance is intrinsically tied to the quality and completeness of the data available in the IEDB and the relationships encoded in the Neo4j knowledge graph. Furthermore, a significant limitation is that the number of new epitopes for the knowledge graph is not large enough to fully generalize the model’s performance to all novel pathogens. This sparsity of relationships for new or rare pathogens may limit the context vectors’ performance in those specific cases. However, this is not a fundamental weakness but rather a clear direction for future work, as the model’s performance can be iteratively improved with the continuous addition of new data to the knowledge graph.

## 4 Conclusion

This manuscript introduces a novel and powerful framework for the identification of IFN-gamma-inducing epitopes by synergistically integrating an ESM2-based MSCNN model with a Neo4j knowledge graph in a Retrieval-Augmented Generation (RAG) framework. Our approach moves beyond simple sequence analysis by leveraging deep biological context, which results in superior predictive performance, evidenced by an AUC of up to 0.99 compared to baseline models. The key innovations of this work lie in the use of a knowledge graph to weight epitope similarity, which enhances both prediction accuracy and model interpretability, the demonstrated therapeutic potential that enables the rapid prototyping of novel peptides for drug development, and the inherent scalability of the RAG and MSCNN architecture. These factors make it suitable for large-scale genomic and proteomic data sets.

This research effectively bridges the gap between computational modeling and practical biomedical application, offering a scalable, biologically informed solution for precision immunology. Future efforts will be directed toward the experimental validation of generated peptides and the continued expansion of the knowledge graph to support a wider array of therapeutic applications, ultimately advancing the design of next-generation vaccines and immunotherapies.

## Supplementary Material



## Data Availability

Our code and data set can be found on GitHub: https://github.com/s1129108/IFNg_DeepKG

## Notes

Experimental tables and figures including ROC curves, charts, t-SNE plots, Grad-CAM, SHAP, and knowledge-graph visualizations (PDF)
