# Supplementary material for: IFNg_DeepKG: A Novel Model for Identifying Interferon-Gamma-Inducing Epitopes Using Knowledge Graph RAG in Biomedical Applications
Source: J Chem Inf Model. 2025 Dec 31;66(1):770–84. doi: 10.1021/acs.jcim.5c02248 (PMC12801304; doi:10.1021/acs.jcim.5c02248)
Supplement: Supplementary file 1 [file ci5c02248_si_001.pdf]

SUPPORTING INFORMATION

IFNg_DeepKG: A Novel Model for Identifying Interferon-Gamma Inducing Epitopes Using Knowledge Graph RAG in Biomedical Applications

Van The Le[1], Juan Peter Timothy Yuune[1], Yu-Yen Ou[1,2*]
[1] Department of Computer Science and Engineering, Yuan Ze University, Chung-Li, 32003, Taiwan
[2] Graduate Program in Biomedical Informatics, Yuan Ze University, Chung-Li, 32003, Taiwan

*Corresponding author: yien@saturn.yzu.edu.tw

CONTENTS

- Experimental result tables (Tables S1-S9)
- Performance charts (Figures S1-S6)
- ROC curves (Figure S7)
- t-SNE visualizations (Figure S8)
- Grad-CAM heatmaps (Figures S9, S10)
- SHAP feature-importance plots (Figures S11, S12)
- Knowledge-graph visualization (Figure S13)

TABLES

Table S1: Performance of using single window size for Human and Mouse training datasets.

Set	Window	Sens	Spec	Acc	MCC	AUC	F1	Pre
H_IFNgTrain	2	0.7510	0.7936	0.7723	0.5458	0.8512	0.7673	0.7855
	4	0.7458	0.8211	0.7834	0.5685	0.8628	0.7749	0.8065
	6	0.7733	0.8155	0.7943	0.5899	0.8705	0.7899	0.8082
	**8**	0.7693	0.8238	0.7965	0.5944	0.8703	0.7908	0.8141
	10	0.7649	0.8213	0.7932	0.5875	0.8667	0.7872	0.8112
	12	0.7514	0.8300	0.7907	0.5832	0.8632	0.7821	0.8156
	14	0.7481	0.8236	0.7859	0.5738	0.8581	0.7774	0.8098
M_IFNgTrain	2	0.7197	0.7520	0.7356	0.4721	0.8074	0.7312	0.7438
	4	0.7279	0.7535	0.7408	0.4820	0.8113	0.7373	0.7476
	**6**	0.7312	0.7527	0.7418	0.4841	0.8128	0.7389	0.7474
	8	0.7172	0.7528	0.7351	0.4709	0.8041	0.7302	0.7447
	10	0.7151	0.7388	0.7269	0.4548	0.7937	0.7234	0.7335
	12	0.7035	0.7492	0.7265	0.4532	0.7933	0.7199	0.7373
	14	0.6801	0.7661	0.7229	0.4480	0.7827	0.7104	0.7443

Table S2: Performance of using multiple window combinations for Human and Mouse training datasets.

Set	Window	Sen	Spec	Acc	MCC	AUC	F1	Pre
H_IFNgTrain	2 4	0.7574	0.8263	0.7919	0.5851	0.8685	0.7844	0.8135
	2 4 6	0.7837	0.8345	0.8091	0.6193	0.8822	0.8041	0.8260
	**2 4 6 8**	0.7801	0.8472	0.8136	0.6288	0.8854	0.8071	0.8364
	2 4 6 8 10	0.7868	0.8357	0.8112	0.6232	0.8837	0.8065	0.8272
	2 4 6 8 10 12	0.8015	0.8216	0.8116	0.6232	0.8830	0.8096	0.8180
	2 4 6 8 10 12 14	0.7728	0.8420	0.8074	0.6163	0.8789	0.8005	0.8303
M_IFNgTrain	2 4	0.7393	0.7499	0.7446	0.4895	0.8179	0.7431	0.7476
	**2 4 6**	0.7191	0.7818	0.7500	0.5026	0.8196	0.7416	0.7679
	2 4 6 8	0.7256	0.7724	0.7489	0.4989	0.8199	0.7427	0.7615
	2 4 6 8 10	0.7326	0.7620	0.7471	0.4953	0.8195	0.7431	0.7552
	2 4 6 8 10 12	0.7353	0.7558	0.7453	0.4913	0.8180	0.7427	0.7507
	2 4 6 8 10 12 14	0.7100	0.7710	0.7405	0.4824	0.8128	0.7322	0.7568

Table S3: Performance of using different filters for Human and Mouse training datasets.

Set	Filter	Sens	Spec	Acc	MCC	AUC	F1	Pre
H_IFNgTrain	64	0.7520	0.8406	0.7962	0.5951	0.8720	0.7867	0.8253
	128	0.7866	0.8262	0.8064	0.6135	0.8806	0.8025	0.8193

	256	0.7801	0.8472	0.8136	0.6288	0.8854	0.8071	0.8364
	512	0.7874	0.8485	0.8180	0.6372	0.8883	0.8122	0.8387
	**1024**	0.7948	0.8455	0.8202	0.6414	0.8889	0.8155	0.8376
	2048	0.7992	0.8346	0.8169	0.6343	0.8883	0.8136	0.8287
M_IFNgTrain	64	0.7176	0.7603	0.7391	0.4792	0.8068	0.7330	0.7505
	128	0.7332	0.7626	0.7477	0.4964	0.8202	0.7438	0.7558
	256	0.7191	0.7818	0.7500	0.5026	0.8196	0.7416	0.7679
	512	0.7186	0.7969	0.7577	0.5181	0.8291	0.7476	0.7809
	**1024**	0.7433	0.7756	0.7594	0.5192	0.8314	0.7555	0.7682
	2048	0.7384	0.7720	0.7553	0.5113	0.8283	0.7511	0.7650

Table S4: Performance of using different hidden units for Human and Mouse training datasets.

Set	Hidden	Sens	Spec	Acc	MCC	AUC	F1	Pre
H_IFNgTrain	**500**	0.7948	0.8455	0.8202	0.6414	0.8889	0.8155	0.8376
	1000	0.7923	0.8462	0.8192	0.6396	0.8903	0.8142	0.8376
	1500	0.7963	0.8441	0.8201	0.6412	0.8903	0.8157	0.8364
M_IFNgTrain	**500**	0.7433	0.7756	0.7594	0.5192	0.8314	0.7555	0.7682
	1000	0.7447	0.7671	0.7559	0.5128	0.8298	0.7529	0.7627
	1500	0.7417	0.7717	0.7571	0.5145	0.8289	0.7532	0.7660

Table S5: Performance of using different learning rates for Human and Mouse training datasets.

Set	Rate	Sens	Spec	Acc	MCC	AUC	F1	Pre
H_IFNgTrain	$1e^{-2}$	0.7705	0.8256	0.7981	0.5975	0.8739	0.7923	0.8162
	$\mathbf{1e^{-3}}$	0.7948	0.8455	0.8202	0.6414	0.8889	0.8155	0.8376
	$1e^{-4}$	0.7714	0.8189	0.7951	0.5911	0.8719	0.7901	0.8101
	$3e^{-4}$	0.7827	0.8365	0.8096	0.6202	0.8828	0.8044	0.8273
	$5e^{-4}$	0.7806	0.8454	0.8130	0.6273	0.8845	0.8067	0.8347
M_IFNgTrain	$1e^{-2}$	0.7172	0.7635	0.7404	0.4814	0.8090	0.7341	0.7522
	$\mathbf{1e^{-3}}$	0.7433	0.7756	0.7594	0.5192	0.8314	0.7555	0.7682
	$1e^{-4}$	0.6918	0.7355	0.7141	0.4286	0.7825	0.7074	0.7248
	$3e^{-4}$	0.7164	0.7854	0.7512	0.5039	0.8225	0.7421	0.7707
	$5e^{-4}$	0.7421	0.7639	0.7528	0.5063	0.8255	0.7500	0.7588

Table S6: Performance of using different batch sizes for Human and Mouse training datasets.

Set	Batch	Sens	Spec	Acc	MCC	AUC	F1	Pre
H_IFNgTrain	64	0.7780	0.8485	0.8133	0.6281	0.8857	0.8064	0.8371
	128	0.7835	0.8464	0.8150	0.6314	0.8868	0.8089	0.8363
	**256**	0.7948	0.8455	0.8202	0.6414	0.8889	0.8155	0.8376
	512	0.7885	0.8357	0.8120	0.6249	0.8827	0.8075	0.8276
M_IFNgTrain	64	0.7287	0.7763	0.7522	0.5059	0.8249	0.7461	0.7656
	128	0.7373	0.7675	0.7520	0.5054	0.8240	0.7480	0.7606
	**256**	0.7433	0.7756	0.7594	0.5192	0.8314	0.7555	0.7682
	512	0.7318	0.7692	0.7506	0.5020	0.8247	0.7456	0.7609

Table S7: Performance with and without IFN-gamma-inducing status for Human and Mouse training datasets.

Set	Weight	Acc	MCC	AUC	F1
H_IFNgTrain	With ifng	0.9465 ± 0.0015	0.8938 ± 0.0032	0.9840 ± 0.0009	0.9475 ± 0.0016
	No ifng	0.9348 ± 0.0016	0.8703 ± 0.0033	0.9778 ± 0.0010	0.9360 ± 0.0016
M_IFNgTrain	With ifng	0.8705 ± 0.0031	0.7412 ± 0.0061	0.9378 ± 0.0021	0.8709 ± 0.0026
	No ifng	0.8631 ± 0.0047	0.7263 ± 0.0095	0.9324 ± 0.0036	0.8629 ± 0.0050

Table S8: Comparison of model performance between ESM2 and Knowledge Graph RAG-enhanced variant in training and independent test datasets.

Set	Feature	Sens	Spec	Acc	MCC	AUC	F1	Pre
5-fold cross validation								
H_IFNgTrain	ESM2	0.7948	0.8455	0.8202	0.6414	0.8889	0.8155	0.8376
	**RAGKG-ESM2**	0.9524	0.9173	0.9348	0.8703	0.9778	0.9360	0.9202
M_IFNgTrain	ESM2	0.7433	0.7756	0.7594	0.5192	0.8314	0.7555	0.7682
	**RAGKG-ESM2**	0.8615	0.8647	0.8631	0.7263	0.9324	0.8629	0.8645
Independent Test 1								
H_IFNgInd1	ESM2	0.7780	0.8686	0.8233	0.6492	0.8909	0.8149	0.8555
	**RAGKG-ESM2**	0.9780	0.9464	0.9622	0.9249	0.9878	0.9628	0.9481
M_IFNgInd1	ESM2	0.7262	0.7776	0.7519	0.5044	0.8174	0.7453	0.7655
	**RAGKG-ESM2**	0.8528	0.9142	0.8835	0.7684	0.9510	0.8798	0.9085
Independent Test 2								
H_IFNgInd2	ESM2	0.7381	0.6580	0.6685	0.2732	0.7548	0.3677	0.2448
	**RAGKG-ESM2**	0.9392	0.8371	0.8504	0.5932	0.9449	0.6212	0.4640
M_IFNgInd2	ESM2	0.6429	0.6095	0.6150	0.1886	0.6688	0.3538	0.2441
	**RAGKG-ESM2**	0.8847	0.8362	0.8442	0.5932	0.9309	0.6505	0.5144

Table S9: Predictions of epitope candidates relevant to Alzheimer's, Diabetes and COVID-19 diseases.

Epitope ID	Host	True label	Predicted label
7493	Human	IFN-gamma Inducing	IFN-gamma Inducing
103041	Human	IFN-gamma Inducing	IFN-gamma Inducing
104630	Mouse	IFN-gamma Inducing	IFN-gamma Inducing
102639	Human	IFN-gamma Inducing	IFN-gamma Inducing
102926	Mouse	IFN-gamma Inducing	IFN-gamma Inducing

FIGURES

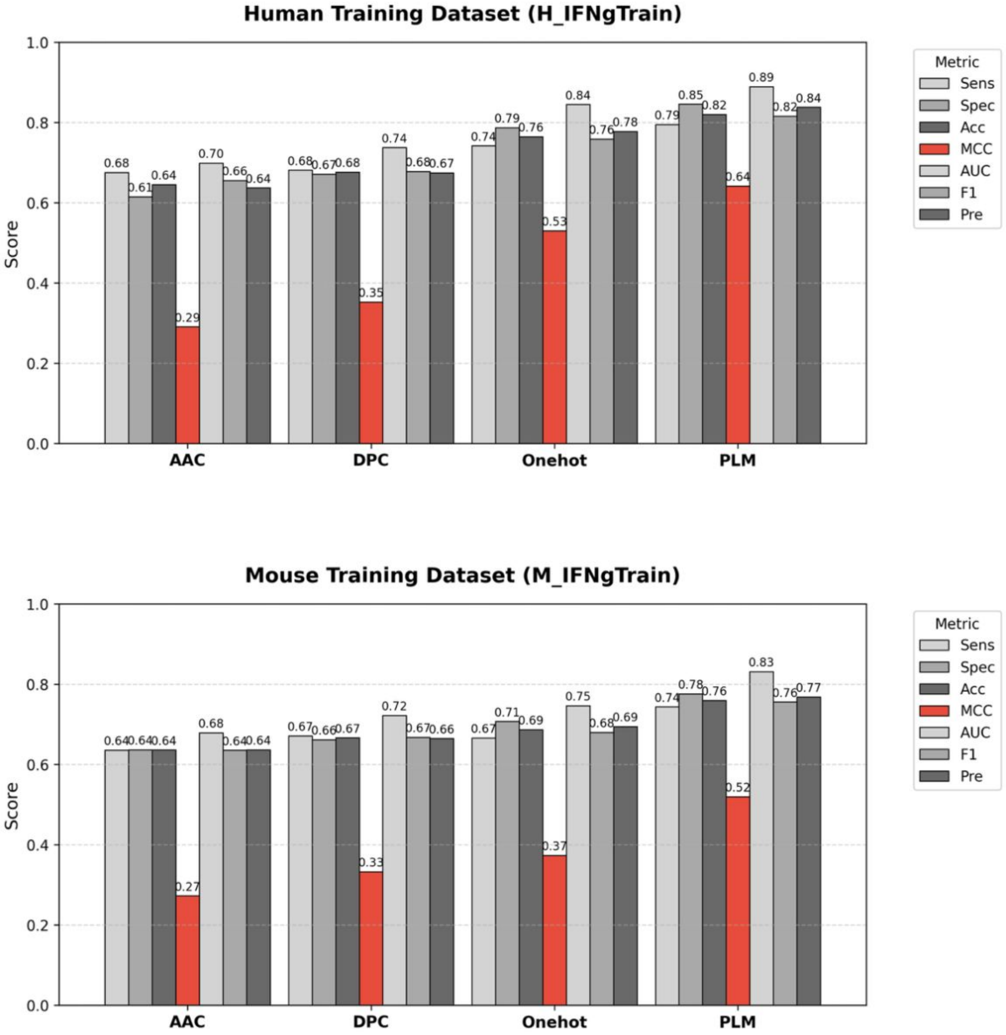

Figure S1: Comparison of model performance using Pretrained Language Model (PLM) embeddings versus traditional sequence features (AAC, DPC, Onehot) on the human (top) and mouse (bottom) training datasets. Each group of bars represents performance across seven evaluation metrics from 5-fold cross-validation. PLM consistently outperforms all traditional features, with the most substantial improvements observed in MCC, AUC , and F1.

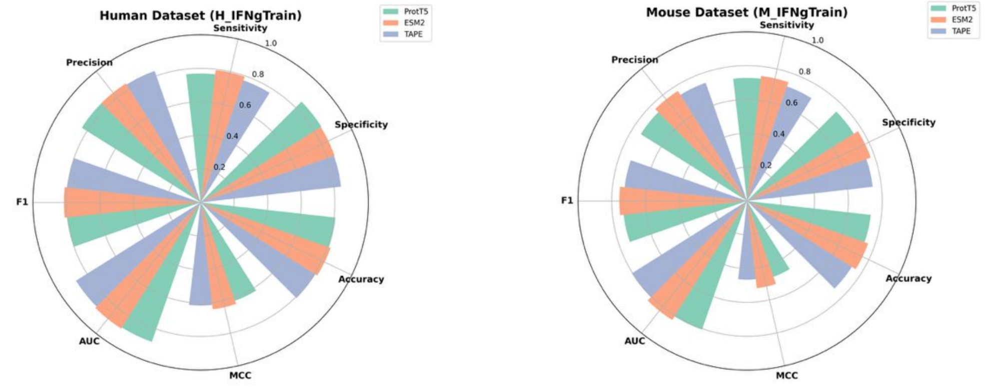

A B

Figure S2: Radar plots illustrating the performance of different pre-trained language models on two training datasets. (A) Human Training dataset (H_IFNgTrain). (B) Mouse Training dataset (M_IFNgTrain). Across both datasets, ESM2 consistently outperforms ProtT5 and TAPE in nearly all evaluation metrics, demonstrating its superior feature representation capability for downstream classification tasks.

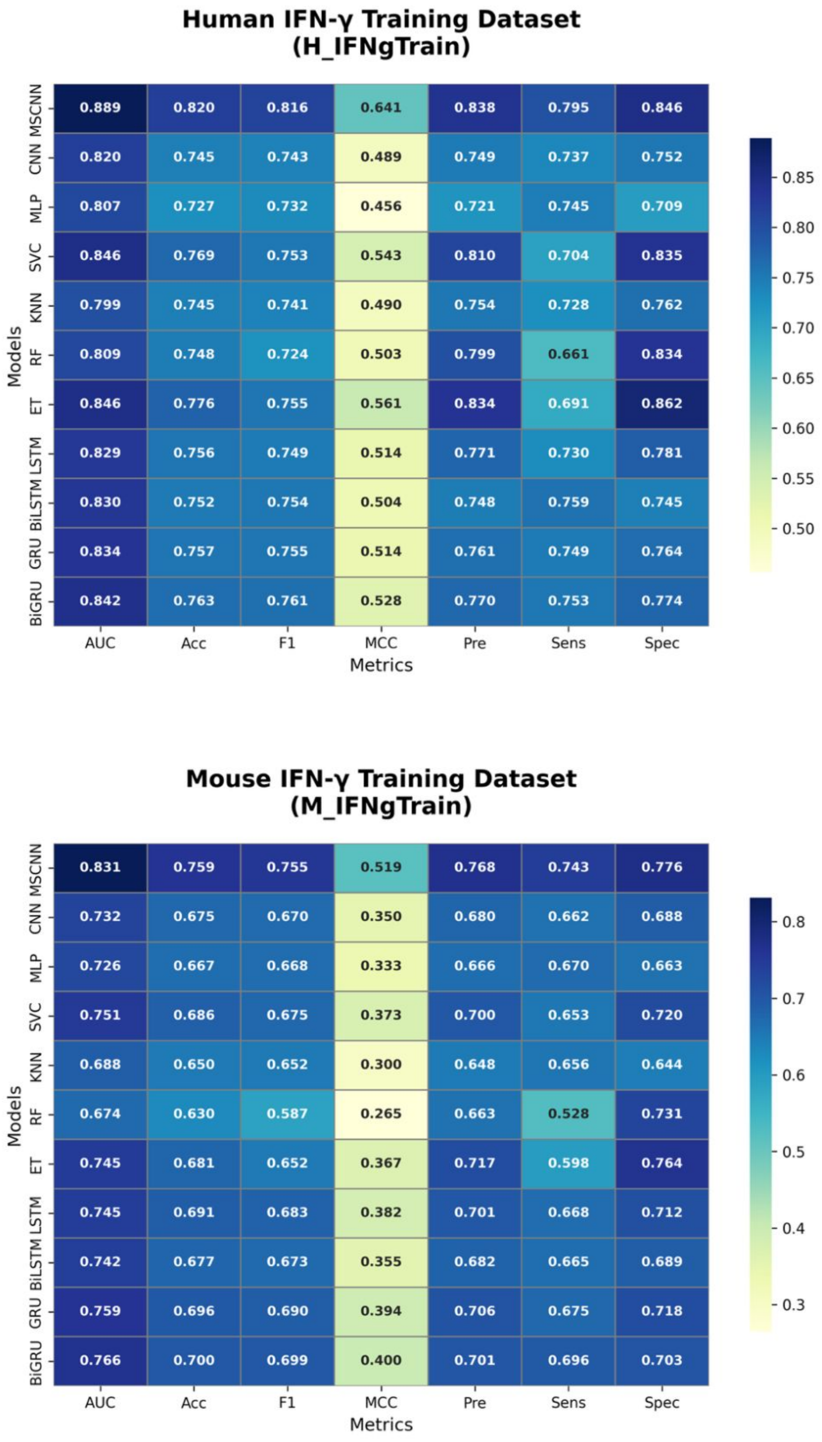

Figure S3: Heatmap comparison of predictive performance across seven evaluation metrics for MSCNN and eleven baseline classifiers using ESM2 embeddings on the human (top) and mouse (bottom) training datasets. Values represent mean performance from 5-fold cross-validation. MSCNN consistently achieves the highest scores across all metrics in both hosts, with particularly pronounced gains in MCC, AUC, and F1, demonstrating the effectiveness of its multi-scale convolutional architecture for IFN-gamma-inducing epitope classification.

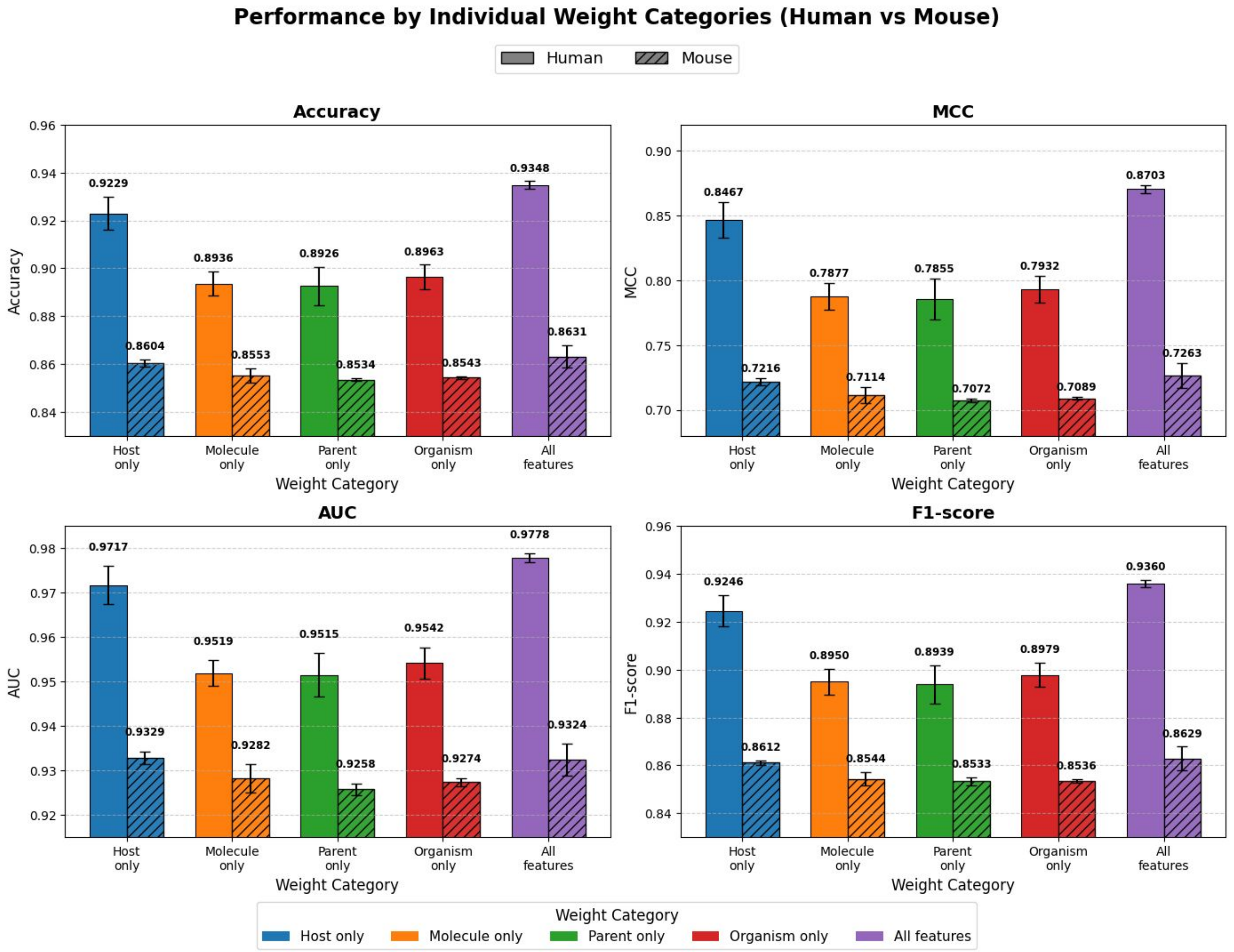

Figure S4: Impact of individual weight categories on model performance. Grouped bar chart showing Accuracy, Matthews Correlation Coefficient (MCC), AUC, and F1-score for Human (solid bars) and Mouse (hatched bars) training datasets. Five weight strategies are compared: Host only, Molecule only, Parent only, Organism only, and All features combined. Error bars represent standard deviation across cross-validation folds. The "All features" model consistently outperforms any single-weight ablation in both species, with the largest gains observed in Human datasets.

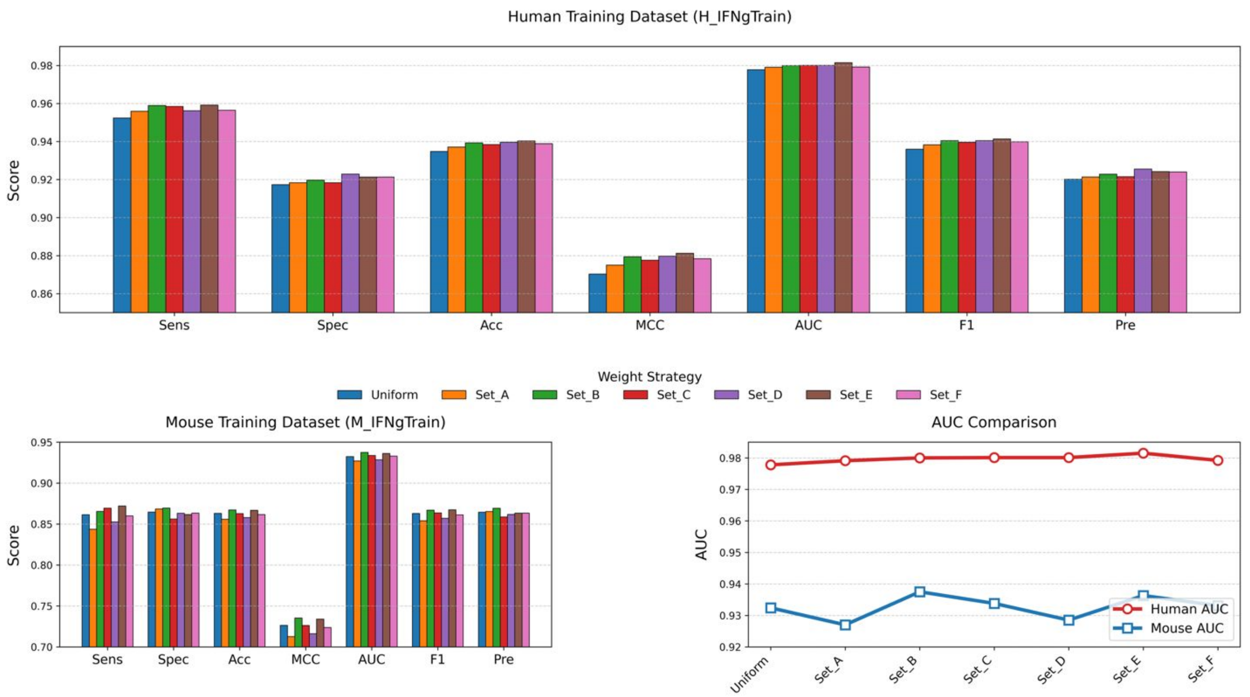

Figure S5: Performance comparison of different weighting strategies on the training datasets. In the H_IFNgTrain dataset, Set E achieves the highest overall performance across evaluation metrics, while in the M_IFNgTrain dataset, Set B demonstrates the best results. Bottom right subplot illustrates the AUC trends in two datasets. Weight strategies include: 'Uniform': [1.0, 1.0, 1.0, 1.0], 'Set_A': [1.0, 0.9, 0.7, 0.8], 'Set_B': [1.0, 0.6, 0.4, 0.5], 'Set_C': [1.0, 0.3, 0.1, 0.2], 'Set_D': [1.0, 0.8, 0.7, 0.9], 'Set_E': [1.0, 0.5, 0.4, 0.6], 'Set_F': [1.0, 0.2, 0.1, 0.3]. Relationship order = ['host', 'source_molecule', 'molecule_parent', 'source_organism'].

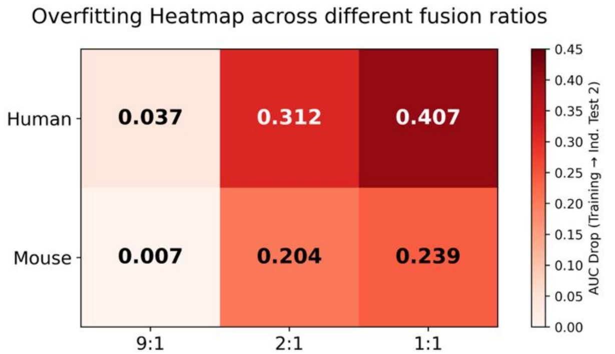

Figure S6: Heatmap illustrating the decline in AUC from training to independent test set 2 (H_IFNgInd2 and M_IFNgInd2) for different query:context fusion ratios. Intensity reflects the magnitude of performance loss. Ratios of 2:1 and 1:1 exhibit pronounced overfitting, whereas the 9:1 ratio preserves generalization across both species.

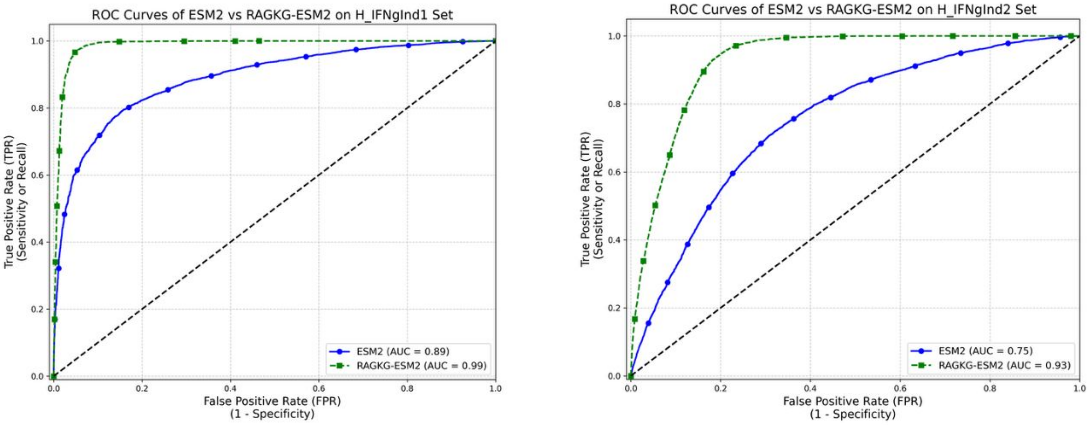

A

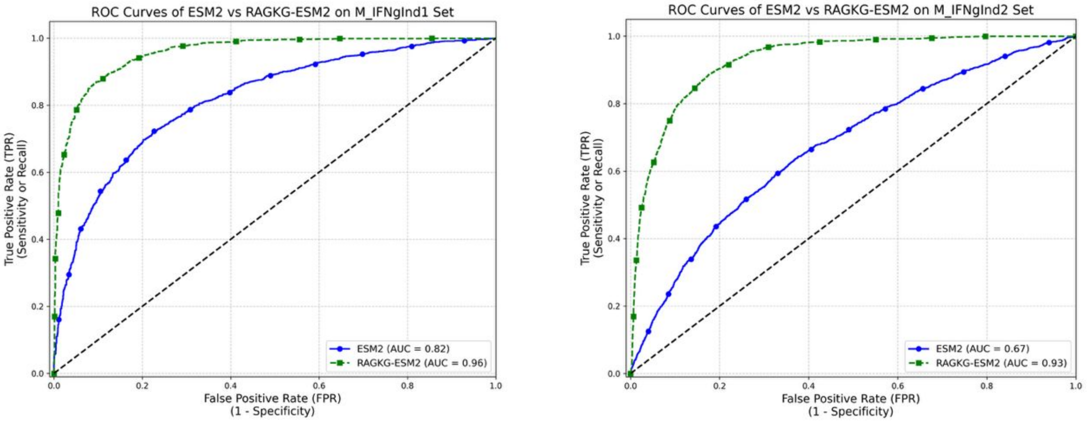

B

Figure S7: AUC-ROC curves of using MSCNN on ESM2 and its Knowledge Graph RAG-enhanced features for training datasets. (A) Human Independent Datasets (H_IFNgInd1 and H_IFNgInd2). (B) Mouse Independent Datasets (M_IFNgInd1 and M_IFNgInd2).

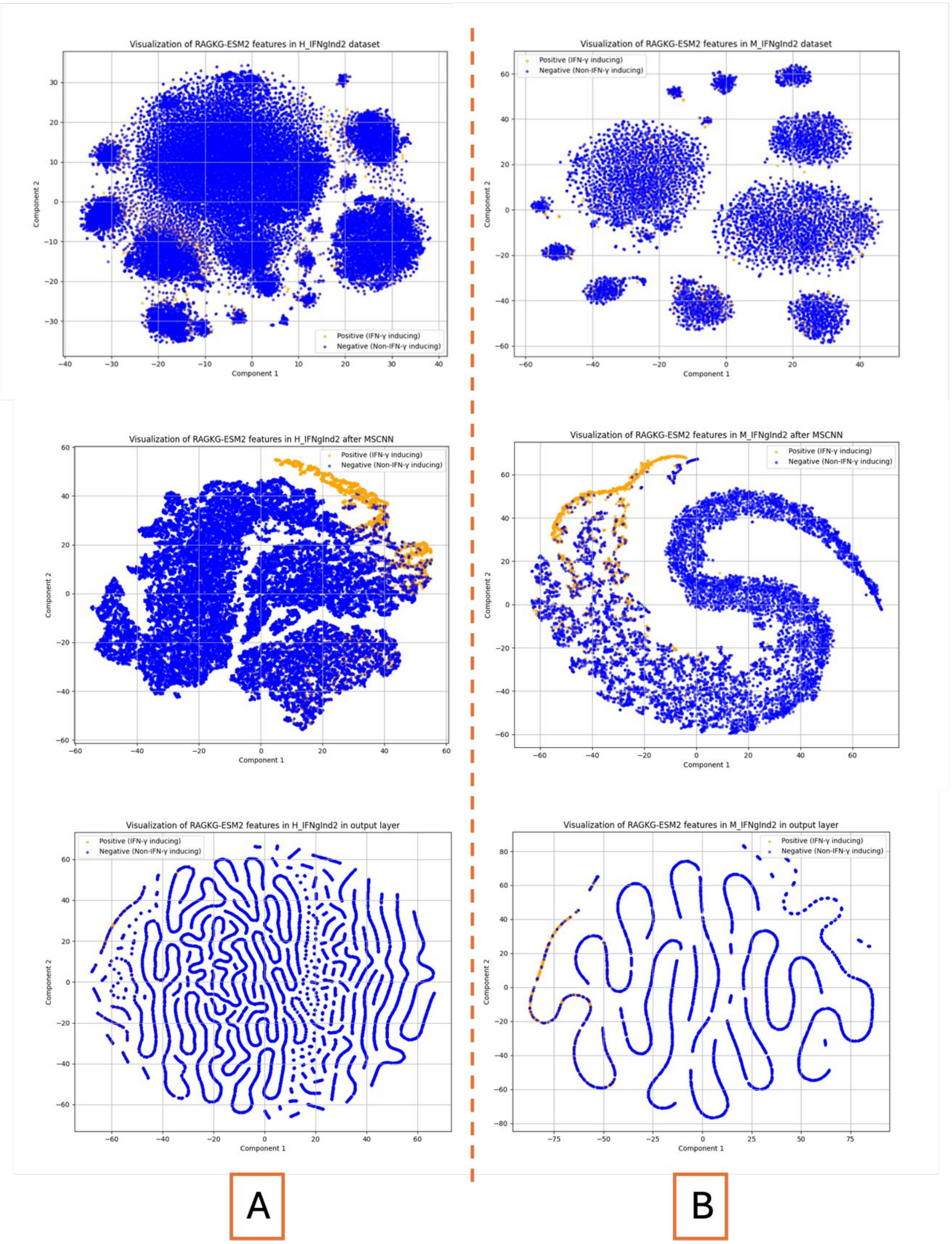

Figure S8: Visualization of Independent Datasets 2 using the MSCNN model. (A) Human host dataset (H_IFNgInd2). (B) Mouse host dataset (M_IFNgInd2). Each panel illustrates the distribution of embeddings at three stages: before processing by the MSCNN model, after feature transformation within the MSCNN, and at the final output layer.

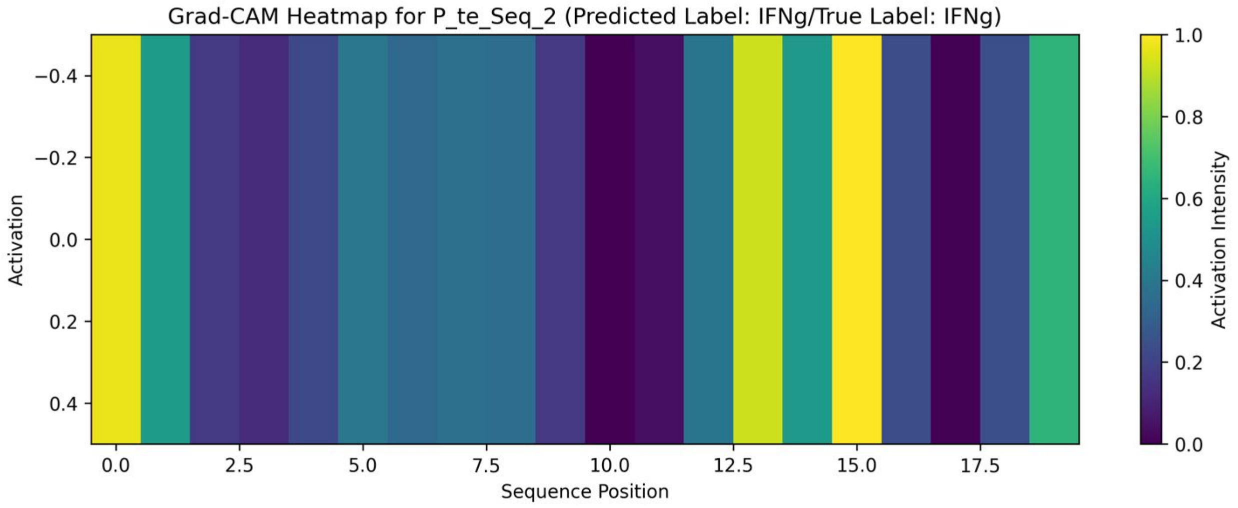
Grad-CAM Heatmap for P_te_Seq_2 (Predicted Label: IFNg/True Label: IFNg)
Activation
−0.4
−0.2
0.0
0.2
0.4
0.0
2.5
5.0
7.5
10.0
12.5
15.0
17.5
Sequence Position
1.0
0.8
0.6
0.4
0.2
0.0
Activation Intensity

A

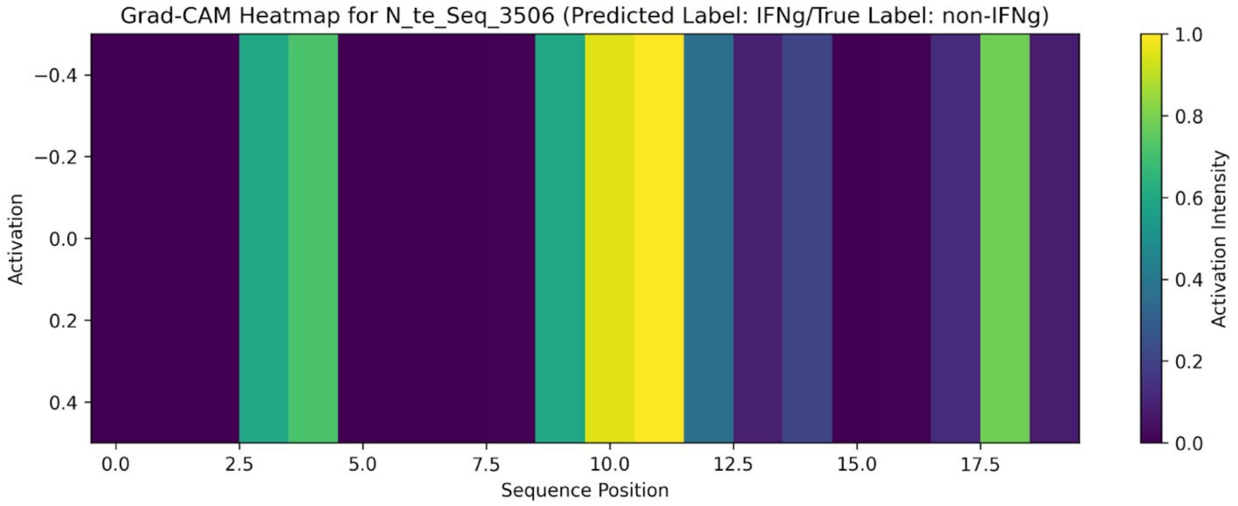
Grad-CAM Heatmap for N_te_Seq_3506 (Predicted Label: IFNg/True Label: non-IFNg)
Activation
−0.4
−0.2
0.0
0.2
0.4
0.0
2.5
5.0
7.5
10.0
12.5
15.0
17.5
Sequence Position
1.0
0.8
0.6
0.4
0.2
0.0
Activation Intensity

B

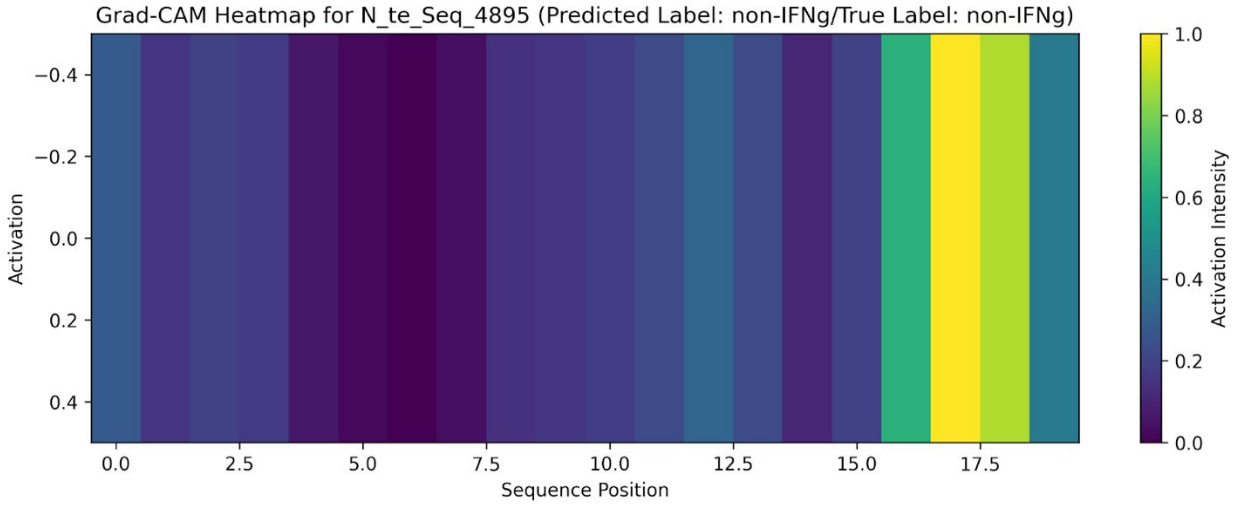
Grad-CAM Heatmap for N_te_Seq_4895 (Predicted Label: non-IFNg/True Label: non-IFNg)
Activation
−0.4
−0.2
0.0
0.2
0.4
0.0
2.5
5.0
7.5
10.0
12.5
15.0
17.5
Sequence Position
1.0
0.8
0.6
0.4
0.2
0.0
Activation Intensity

C

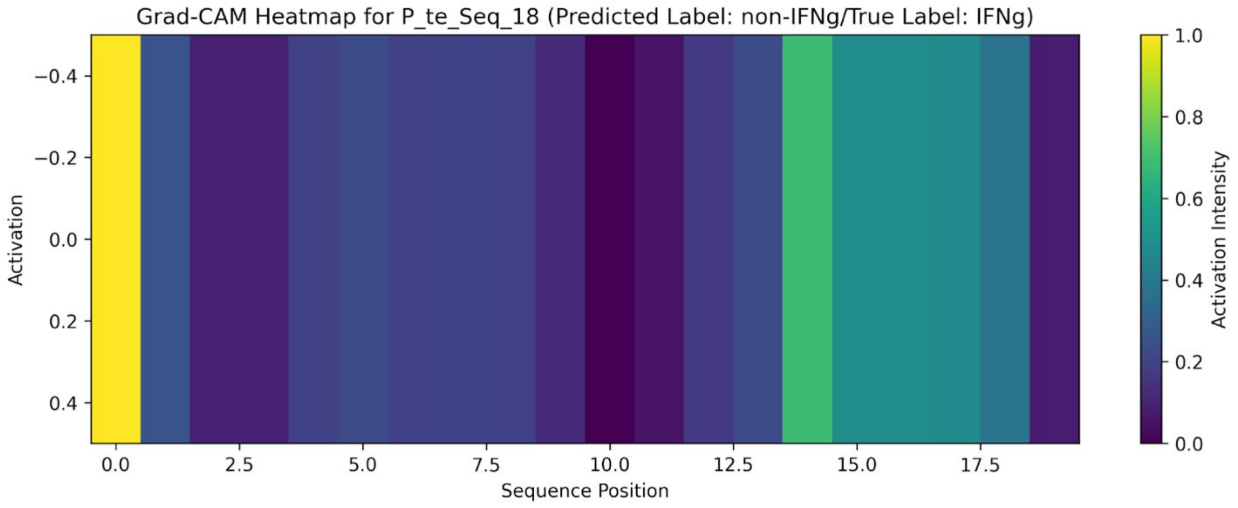

D

Figure S9: Grad-CAM visualizations of representative proteins from the Independent Dataset 1 H_IFNgInd1. (A) P_te_Seq_2 (LPRQRAYL): correctly classified IFN-gamma-inducing epitope showing strong activation domains. (B) N_te_Seq_3506 (MIEEIDADGSGTVDF): false-positive IFN-gamma-inducing epitope prediction caused by spurious activation on a glycine-rich hydrophobic motif. (C)N_te_Seq_4895 (VPAKSVCGPVYCFTP): correctly classified non-IFN-gamma-inducing epitope with activation aligned to transmembrane helices. (D)P_te_Seq_18 (HEIHIGYL) : false-negative IFN-gamma-inducing epitope prediction due to.

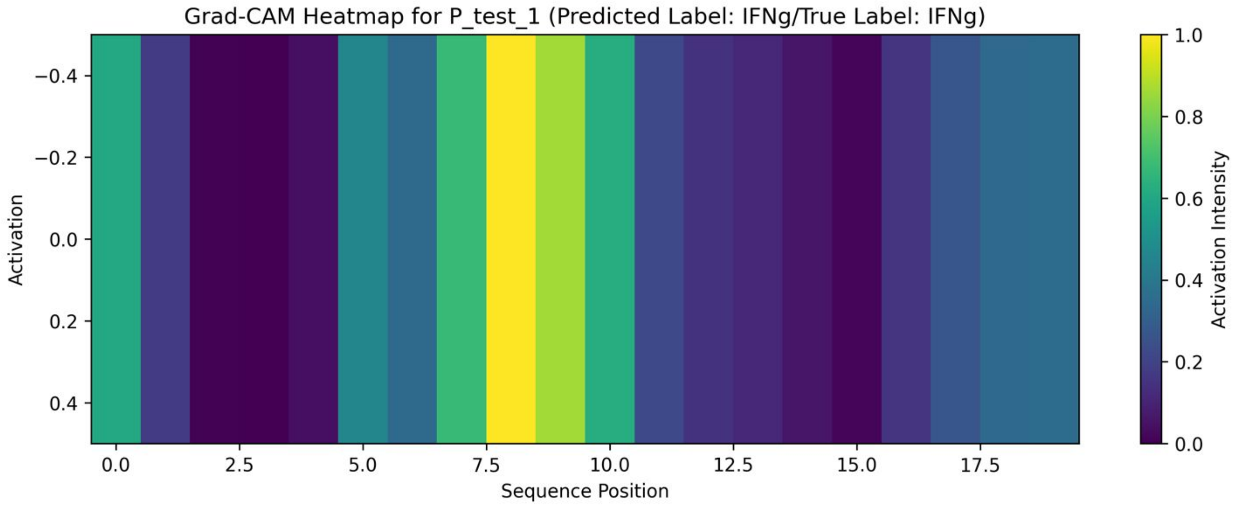

A

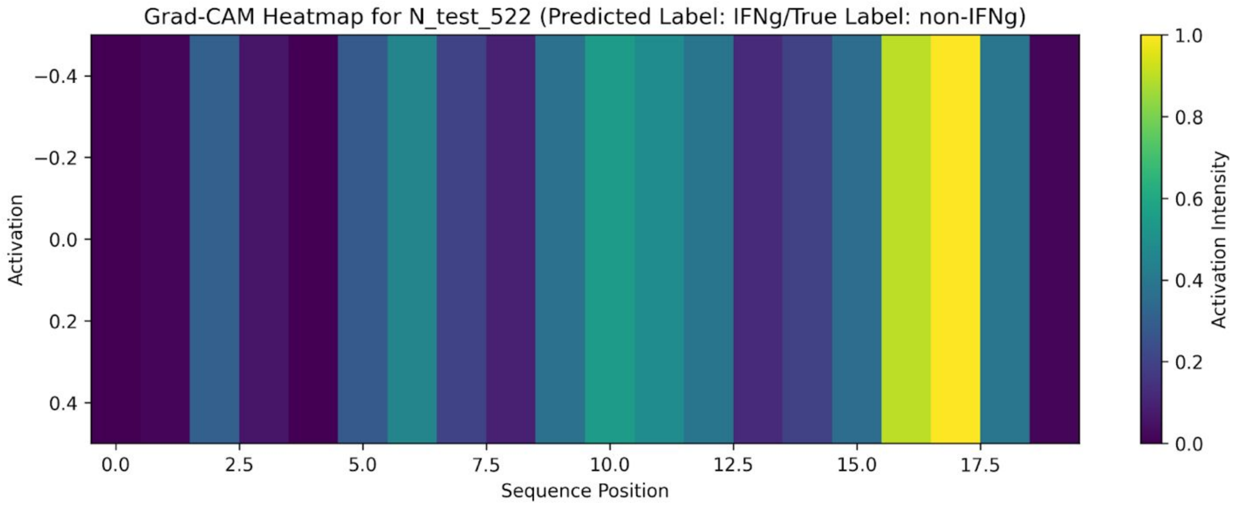
Grad-CAM Heatmap for N_test_522 (Predicted Label: IFNg/True Label: non-IFNg)
Activation
Sequence Position
Activation Intensity
−0.4
−0.2
0.0
0.2
0.4
0.0
2.5
5.0
7.5
10.0
12.5
15.0
17.5
1.0
0.8
0.6
0.4
0.2
0.0

B

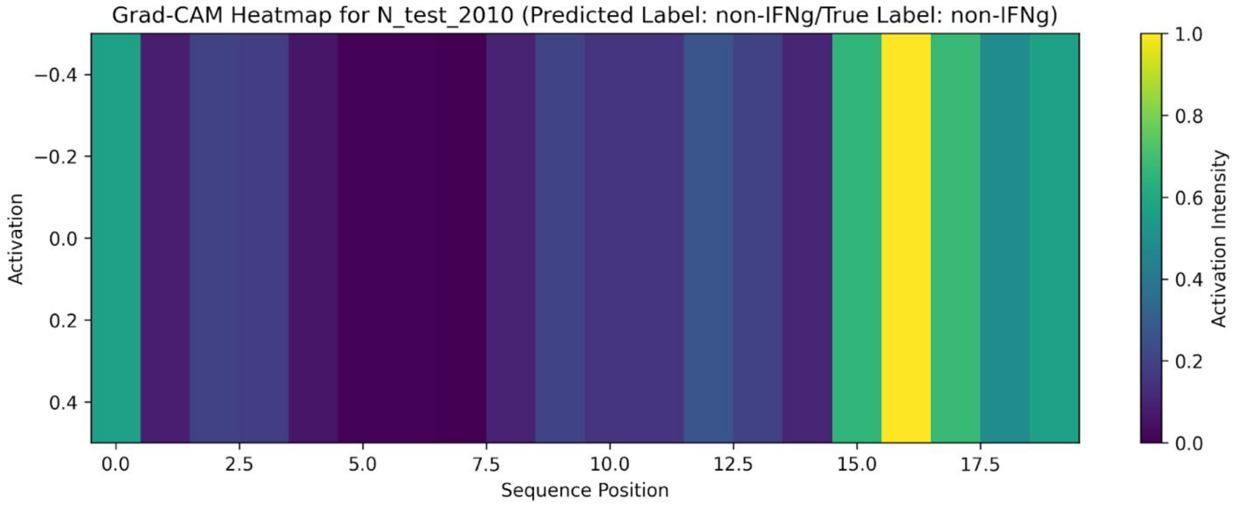
Grad-CAM Heatmap for N_test_2010 (Predicted Label: non-IFNg/True Label: non-IFNg)
Activation
Sequence Position
Activation Intensity
−0.4
−0.2
0.0
0.2
0.4
0.0
2.5
5.0
7.5
10.0
12.5
15.0
17.5
1.0
0.8
0.6
0.4
0.2
0.0

C

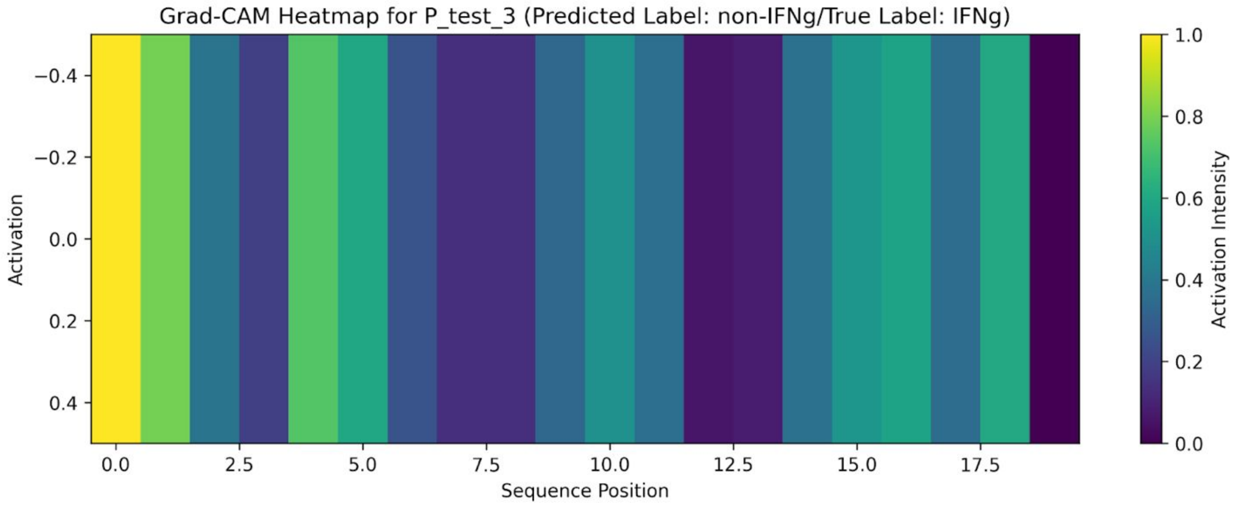
Grad-CAM Heatmap for P_test_3 (Predicted Label: non-IFNg/True Label: IFNg)
Activation
Sequence Position
Activation Intensity
−0.4
−0.2
0.0
0.2
0.4
0.0
2.5
5.0
7.5
10.0
12.5
15.0
17.5
1.0
0.8
0.6
0.4
0.2
0.0

D

Figure S10: Grad-CAM visualizations of representative proteins from the Independent Dataset 1 M_IFNgInd1. (A) P_test_1 (VSVVFAAL): correctly classified IFN-gamma-inducing epitope showing strong activation. (B) N_test_522 (KQTCNSSAV): false-positive IFN-gamma-inducing epitope prediction caused by spurious activation on a local glycine-rich hydrophobic motif. (C) N_test_2010 (TVVNKVLIPM): correctly classified non-IFN-gamma-inducing epitope with activation aligned to transmembrane helices. (D) P_test_3 (LYQLENYC) : false-negative IFN-gamma-inducing epitope prediction due.

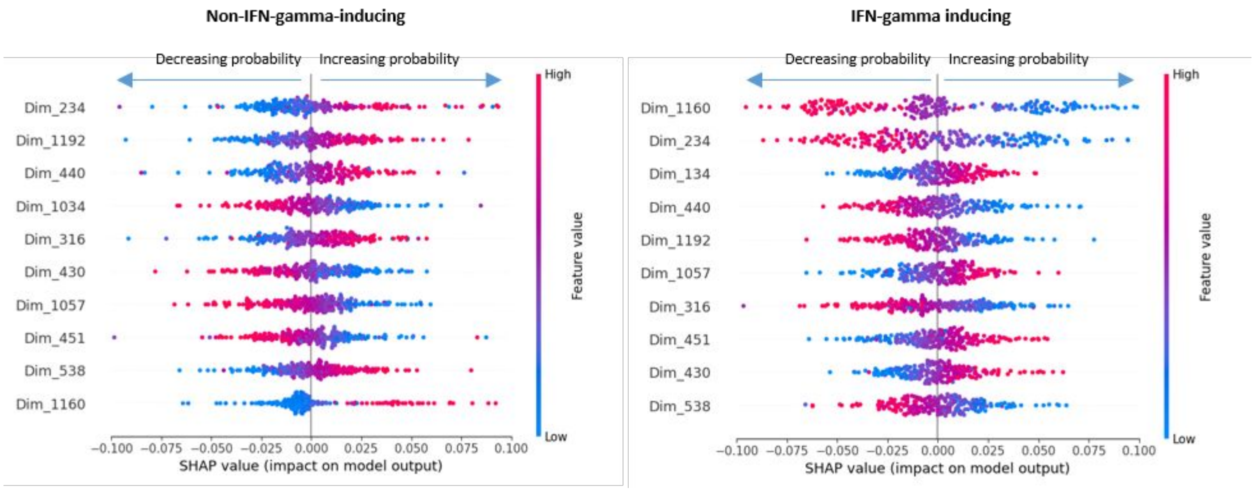

A

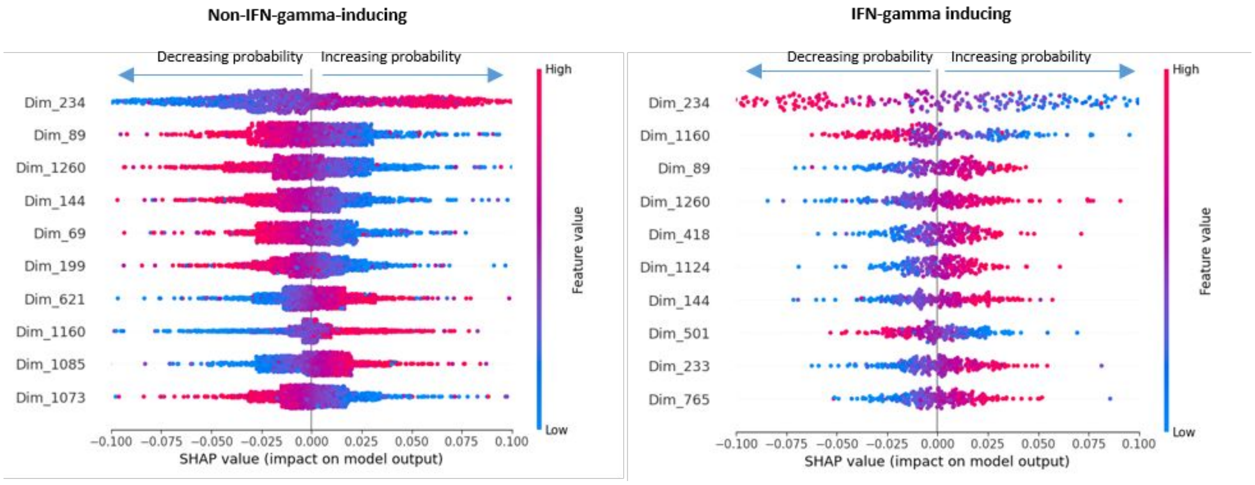

B

Figure S11: Comparative SHAP Summary for the top-10 most important features on the classification of IFN-gamma-inducers (Class 0: Non-IFN-gamma-inducing epitopes; Class 1: IFN-gamma-inducing epitopes) across two Human datasets (H_IFNgInd1 dataset (Panel A) and H_IFNgInd2 (Panel B). In each panel, the plot on the left represents contributions toward Class 0, and the plot on the right represents contributions toward Class 1. Each row is a feature, ranked by its overall importance, and each dot is a sample. The x-axis indicates the SHAP value (impact on model output); values right of zero increase the designated class's probability. The color of the dot denotes the feature value (red = high; blue = low). Across both datasets, Dim_234 is a consistently important predictor. Notably, high Dim_1160 is the strongest predictor for IFN-gamma-inducing

epitopes in Panel A, while high Dim_234 and high Dim_89 are the strongest antagonistic predictors for IFN-gamma-inducing and Non-IFN-gamma-inducing epitopes, respectively, in Panel B.

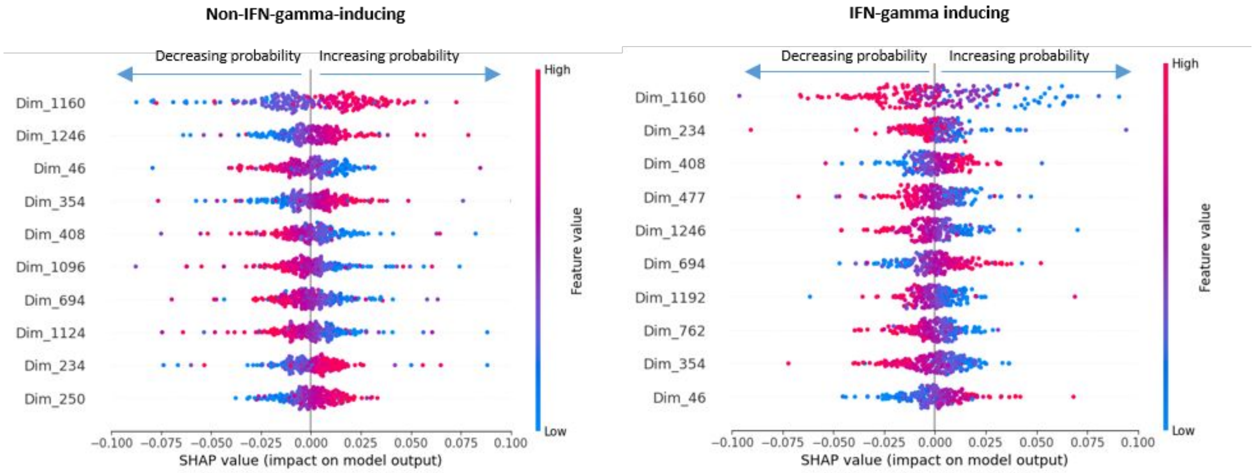

A

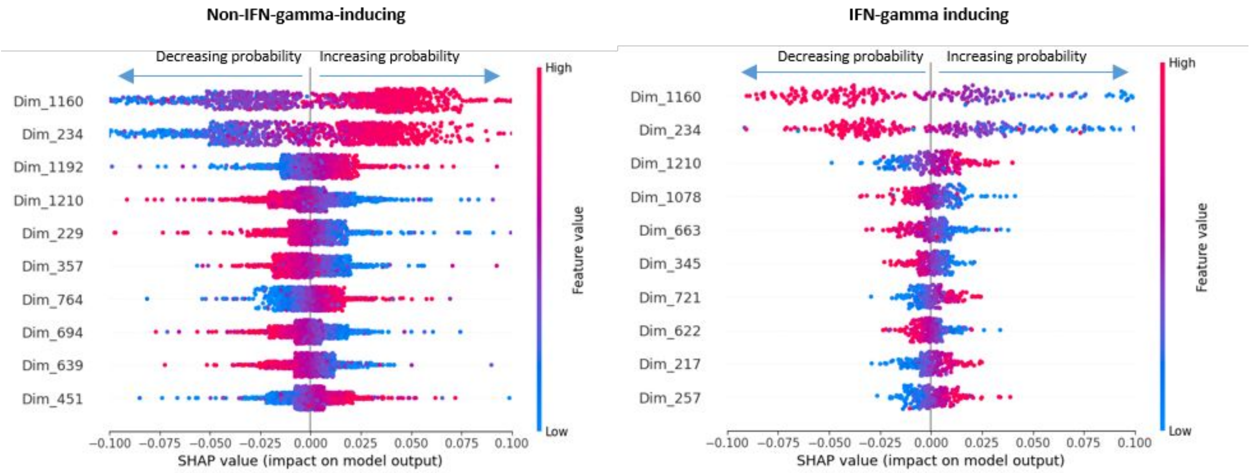

B

Figure S12: Comparative SHAP Summary for the top-10 most important features on the classification of IFN-gamma-inducers (Class 0: Non-IFN-gamma-inducing epitopes; Class 1: IFN-gamma-inducing epitopes) across two Mouse datasets (M_IFNgInd1 dataset (Panel A) and M_IFNgInd2 (Panel B). In each panel, the plot on the left represents contributions toward Class 0, and the plot on the right represents contributions toward Class 1. Each row is a feature, ranked by its overall importance, and each dot is a sample. The x-axis indicates the SHAP value (impact on model output); values right of zero increase the designated class's probability. The color of the dot denotes the feature value (red = high; blue = low). Overall, the dimensions Dim_1160 and Dim_234 are the universally dominant predictors across both cohorts. In Panel B, low Dim_1210 is shown to be a strong positive predictor for the IFN-gamma-inducers.

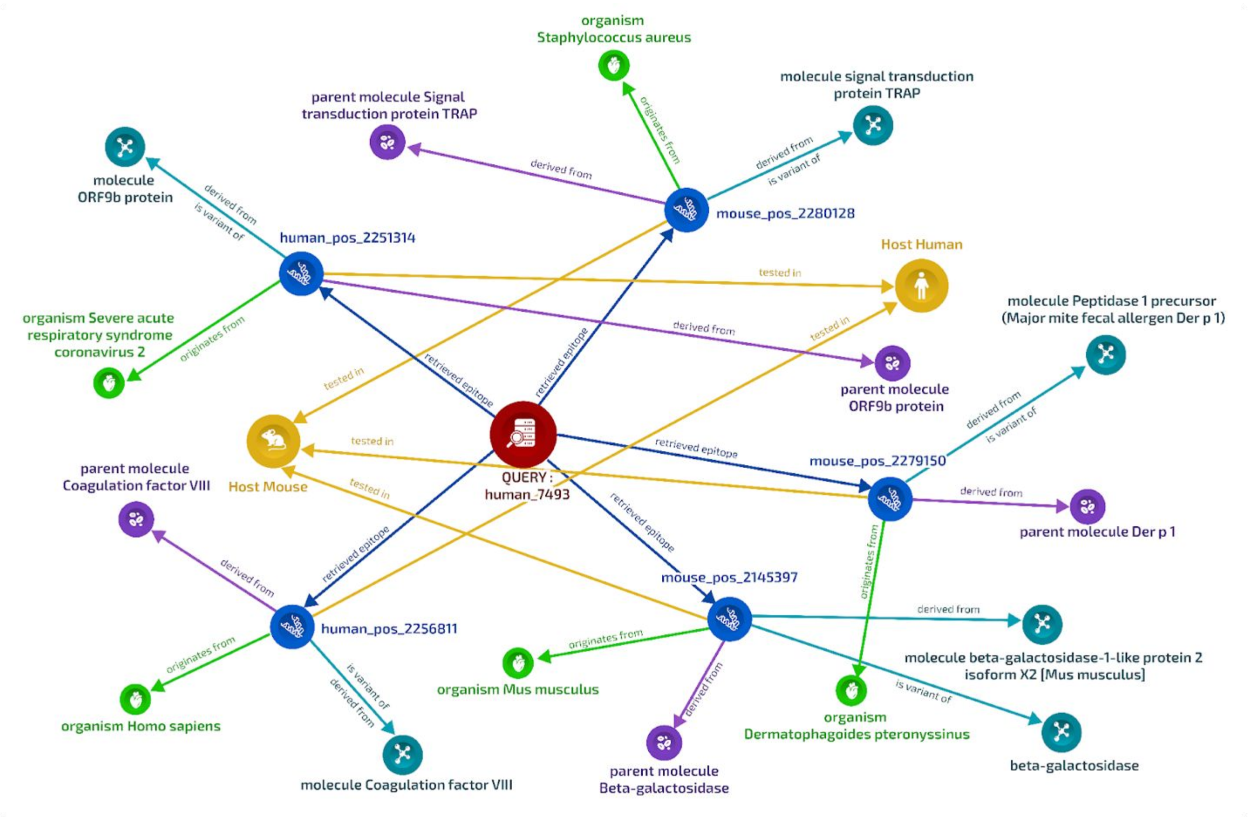

Figure S13: Knowledge graph illustrations of the epitope inducing IFN-gamma (ID: human_7493) in Alzheimer's disease. The relationships collected from five epitope candiates that the test sample retrieved from RAG-KG database.
